# Electrically driven single microwire-based single-mode microlaser

**DOI:** 10.1038/s41377-022-00874-w

**Published:** 2022-06-29

**Authors:** Xiangbo Zhou, Mingming Jiang, Kai Xu, Maosheng Liu, Shulin Sha, Shuiyan Cao, Caixia Kan, Da Ning Shi

**Affiliations:** College of Physics, MIIT Key Laboratory of Aerospace Information Materials and Physics, Key Laboratory for Intelligent Nano Materials and Devices, Nanjing University of Aeronautics and Astronautics, No. 29 Jiangjun Road, Nanjing, 211106 China

**Keywords:** Semiconductor lasers, Microresonators

## Abstract

Engineering the lasing-mode oscillations effectively within a laser cavity is a relatively updated attentive study and perplexing issue in the field of laser physics and applications. Herein, we report a realization of electrically driven single-mode microlaser, which is composed of gallium incorporated zinc oxide microwire (ZnO:Ga MW) with platinum nanoparticles (PtNPs, *d* ~ 130 nm) covering, a magnesium oxide (MgO) nanofilm, a Pt nanofilm, and a p-type GaN substrate. The laser cavity modes could resonate following the whispering-gallery mode (WGM) among the six side surfaces by total internal reflection, and the single-mode lasing wavelength is centered at 390.5 nm with a linewidth of about 0.18 nm. The cavity quality factor *Q* is evaluated to about 2169. In the laser structure, the usage of Pt and MgO buffer layers can be utilized to engineer the band alignment of ZnO:Ga/GaN heterojunction, optimize the p-n junction quality and increase the current injection. Thus, the well-designed device structure can seamlessly unite the electron-hole recombination region, the gain medium, and optical microresonator into the PtNPs@ZnO:Ga wire perfectly. Such a single MW microlaser is essentially single-mode regardless of the gain spectral bandwidth. To study the single-mode operation, PtNPs working as superabsorber can engineering the multimode lasing actions of ZnO:Ga MWs even if their dimensions are typically much larger than that of lasing wavelength. Our findings can provide a straightforward and effective scheme to develop single-mode microlaser devices based on one-dimensional wire semiconductors.

## Abstract (web summary)

An electrically driven single-mode microlaser, containing a lasing peak at 390.5 nm, a narrow FWHM ~ 0.18 nm, and *Q*-factor ~ 2169, was proposed and demonstrated.

## Graphical abstract

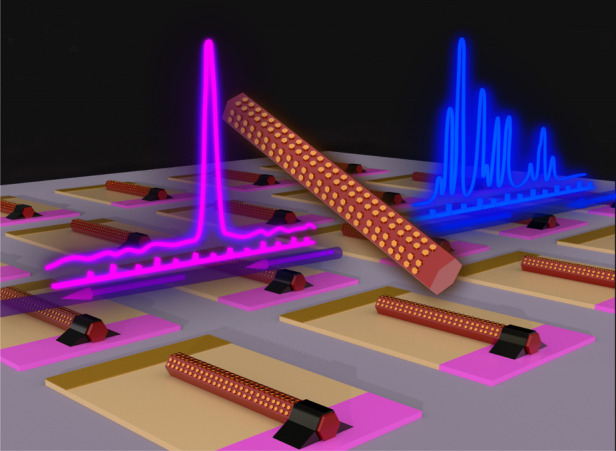

## Introduction

Miniaturization and integration of low-dimensional semiconductor micro/nanowire lasers oscillating at a single frequency have attracted tremendous research interest due to the potential for commercial applications in on-chip optical communication, superresolution imaging, quantum information processing, ultradense data storage, etc^1–7^. In the last few decades, a variety of experimental and theoretical approaches have been proposed to develop single-mode laser devices, such as introducing distributed feedback (DFB) gratings or distributed Bragg reflector (DBR) mirrors, reducing the size of optical cavities, introducing another cavity as a modulator to expand free spectral range (FSR) of the coupled cavities via Vernier effect, inducing parity-time symmetry breaking and so on^8–16^. Among these reported strategies, reducing the length of the microcavity is the most direct and effective solution to realize single-mode laser devices, but at the expense of optical gain and threshold^8^. With the aid of Vernier effect, incorporating another optical resonator as a modulator can be utilized to tune the FSR of coupled cavities offering strong mode selection and competition for single-mode lasing, but the experiment should be operated using fine micromanipulation technology^9^. The introduction of some specific frequency selection mechanisms has been widely utilized to obtain single-mode lasing with the aid of external tools (such as DBR and DFB), but are typically manufactured through expensive microfabrication processes and complex manipulation^10,11^. Further, the development of single-mode semiconductor micro/nanowire laser devices with good monochromaticity (including high-quality factors, narrow linewidths, and low thresholds) remains a critical challenge, especially for the realization upon electrical excitation, which is still a predominant drawback limiting their practical applications.

One-dimensional (1D) wire semiconductors, such as micro/nanowires, nanoribbons, nanotubes and so on, have been extensively researched for constructing light-emitting diodes (LEDs) and laser diodes (LDs) due to their passive waveguides and strong confinement of electrons, holes, and photons^17–22^. The lasing features acquired from previously published 1D wired laser devices are currently multiple modes due to their larger dimensions than that of the lasing wavelengths. Among the mainly reported approaches that have been extensively researched to engineer lasing modes in a laser resonator, reducing the size is the most effective and direct method to obtain single-mode operation. However, it brings about higher laser threshold and optical loss in the laser cavity, especially upon the operation of electrical excitation, which is generally difficult to achieve^14,23,24^. Generally, to engineering the modes oscillating within a laser resonator upon electrically pumped, effective manipulation of cavity resonant modes between gain and loss is critical for a stable single-mode operation in a 1D wire laser. In addition, the fabrication of electrically pumped microlaser devices is restricted by preparing highly-qualified optical microresonators, Ohmic contact between metal electrodes and gain media, high-quality Schottky/p-n junction, and optimized junction interface^25–27^. Therefore, the design of a microlaser device that successfully combining the electron-hole generation, a laser medium, and an optical microresonator within the 1D wire structure is a significant challenge that can enable realization of high-performance lasing oscillation upon electrical excitation. Furthermore, the expected searching for a kind of compact and suitable approach to modulate control the modes oscillating within a laser cavity and obtaining single-mode operation, is still demanding. And the implementation scheme should concern the merits of easy manipulation and control without damaging the optical cavity^5,17,18,24^.

In this work, we proposed and demonstrated a feasible approach to realize a single-mode microlaser. The microlaser device is made of a gallium doped zinc oxide microwire with platinum nanoparticles cladding (PtNPs@ZnO:Ga MW), Pt and magnesium oxide (MgO) buffer layers, and a p-type gallium nitride (GaN) film. Upon continuous-wave (CW) operation of electrical excitation with the injection current well above the lasing threshold, the single-mode lasing peak is clearly observed at a wavelength of 390.5 nm. The lasing linewidth is about 0.18 nm, corresponding to a quality (*Q*) factor of ~2169. The transition changing from spontaneous radiation to amplified stimulated emission, and the unambiguous laser oscillation were observably derived from the dramatic increase of spectral purity of the oscillation mode within the ZnO:Ga MW microresonator. Obtaining the electroluminescence (EL) in the ultraviolet wavelengths, the buffer layers of Pt and MgO were inserted into the n-ZnO:Ga MW/p-GaN heterojunction, leading to appropriately engineering the band alignment of ZnO:Ga/GaN. Thus, the active region of the electron-hole recombination region is primarily distributed in the ZnO:Ga MW even at high injection levels, suggesting the absence or negligible presence of the contributing factors that lowering the efficiency of conventional LEDs and laser diodes containing leakage current, Auger recombination, defect-related recombination, junction temperature influences and so on. In the absence of PtNPs cladding, the lasing peaks acquired from the n-ZnO:Ga/Pt/MgO/p-GaN heterojunction devices exhibit multiple modes with periods over a wide spectral range. The manipulation of large-size PtNPs on cavity resonant modes in the fabricated microlaser device is studied for achieving stable single-mode operation. It is found that PtNPs with diameter *d* ~ 130 nm serving as superabsorbers can enable lasing-mode engineering, achieving single-mode operation. The working principle of the constructed laser device and the mode management in a ZnO:Ga MW laser cavity have been investigated in detail. To the best of our knowledge, the experimental results are the first realization of electrically driven single-mode microlaser at room temperature. These findings are expected to enable an important step toward the workable implementation of single-mode coherent light sources under electrical excitation.

## Results

As previous literature illustrated that, ZnO-based nano-/microstructures can function as optical cavities and support lasing actions, such as WGM, random, Fabry-Perot and so on^28–30^. Nevertheless, the practical application of optically pumped ZnO nano-/microstructures lasers would be of limited significances technologically. In the present research, the preparation of highly-crystallized ZnO:Ga MWs is expected to have a lower potential drop and resistive loss. Thus, the as-grown ZnO:Ga samples are the most likely not the predominant component for developing droop-free LEDs and lasers upon electrical excitation, which have been widely observed in the ZnO/GaN heterojunction emission devices^23,31^. Generally, the achievement of electrically driven lasing actions should require efficient injection of electrons and holes into the optical resonator region. In the case of individual ZnO:Ga MWs, a unambiguous superiority of 1D-based device structures is the potential competence to integrate different high-quality semiconductors as desired to design the required device structures. Alternatively, the fabrication of ZnO-based LEDs and lasers has been realized by combining p-type GaN film working as hole transporting source. The device performances have been subject to the generation occurring at the ZnO/GaN heterointerface, optical loss, Auger recombination, quantum confinement Stack effect and so on^18,32,33^. To figure out these limitations, low-dielectric buffer layer, such as MgO, was inserted into the ZnO/GaN heterostructure. In the presence of MgO buffer interlayer, the energy band alignment of n-ZnO:Ga MW/p-GaN heterojunction could be appropriately manipulated. The benefits of the as-designed n-ZnO:Ga MW/MgO/p-GaN heterojunction is concluded, such as reducing electron leakage, engineering carrier transport path into the wire and lowing the optical loss at the junction interface, thus, achieving typically excitonic electron-hole recombination in the single ZnO:Ga MWs. In the designed n-ZnO:Ga MW/MgO/p-GaN device architecture, the p-type GaN film contact is treated as an effective for the current spreading and for the effective hole injection into the ZnO:Ga samples. The generation of electron-hole recombination occurring in the GaN region is insignificant. However, the used MgO interlayer would give rise to unfavorable characteristics, including a lower current injection, larger turn-on voltage, and lower EL efficiency^22,31,34^.

**Fig. 1:**
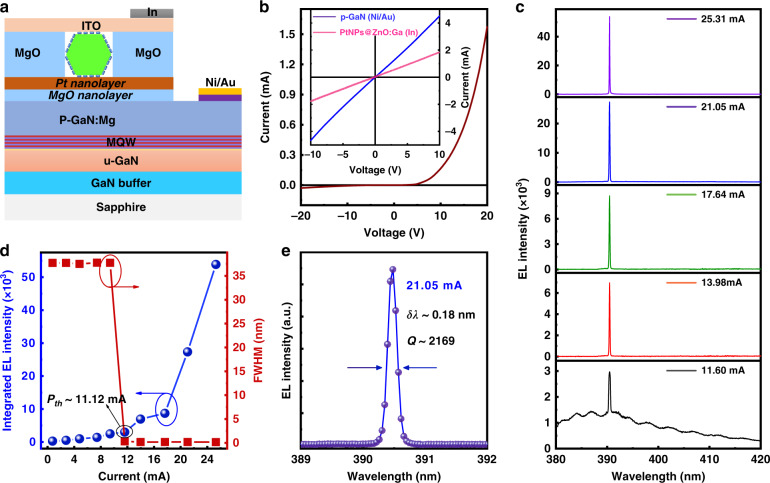
CW operation of EL characterization of as-constructed n-PtNPs@ZnO:Ga MW/Pt/MgO/p-GaN heterojunction device. **a** Schematic illustrating the cross-section of the laser device architecture based on the n-PtNPs@ZnO:Ga MW/Pt/MgO/p-GaN heterojunction. In the device configuration, ITO and Ni/Au working as electrodes are responsible for the current injection. **b** *I–V* curve of the fabricated single MW heterojunction emission device. Inset: the *I–V* curves of Ni/Au electrode contacted to the p-type GaN film, and a single PtNPs@ZnO:Ga MW, respectively. **c** Current-dependent EL spectra of the as-fabricated single MW-based LED, as the injection current is increased from 11.6 to 25.31 mA. **d** Variations of the integrated EL intensity and spectral FWHM as a function of injection current, showing a lasing threshold of 11.12 mA. **e** EL spectrum via Lorentz fitting at the input current of 21.05 mA, providing the FWHM of the lasing peak *δλ* ~ 0.18 nm, and the corresponding *Q*-factor value is calculated to about 2169

To further optimize the junction interface quality, particularly the electrical properties, Pt buffer layer was further deposited on the MgO/p-GaN substrate in this study. The incorporation of the Pt nanolayer may facilitate carrier transport in the as-designed LEDs, and also make the electrical contact between the ZnO:Ga MW and p-type GaN film much easier and more reliable, giving rise to optimizing radiative recombination and charge injection/transport simultaneously in these fabricated emission devices^24^. A schematic of our designed light-emitting device is shown in Fig. 1a, which is composed of a single ZnO:Ga MW covered with PtNPs, a MgO nanofilm, a Pt nanofilm and a p-type GaN substrate. Detailed information on the sample characterization and device fabrication are provided in the Supplementary Materials (Figs. S1–S3 and Table S1, Support information). Figure S2 presents a scanning electron microscopy (SEM) image of the single ZnO:Ga MW covered by PtNPs. The SEM observation reveals that physically isolated PtNPs is uniformly distributed on the side surfaces of ZnO:Ga MW, and the average diameter is approximatively measured to about 130 nm. Figure 1b shows the current-voltage (*I–V*) curve of the fabricated n-PtNPs@ZnO:Ga MW/Pt/MgO/p-GaN structure, which exhibits the typical rectifying characteristics with a turn-on voltage of 5.5 V. Besides, the leakage current could be neglected under reverse bias. The linear *I–V* curve of Ni/Au electrode contacted to the p-type GaN film suggests that a good Ohmic contact has been created at p-type electrodes (the blue solid line shown in the inset of Fig. 1b); while the analysis of *I–V* characteristic of a single PtNPs@ZnO:Ga MW also confirms the well-defined Ohmic contacting behavior (The pink solid line illustration in the inset of Fig. 1b). The as-designed n-PtNPs@ZnO:Ga MW/Pt/MgO/p-GaN heterojunction device revealed excellent rectification characteristics, which is principally because of the insertion of Pt/MgO buffer layers.

In forward biasing, the fabricated 1D wire device structure illustrates strong EL emissions featuring in ultraviolet band, with the light-emitting regions distributed along the wire body functioning as optical waveguides. The EL emission is so bright that can be visibly observed by naked eyes under normal lighting conditions. The emitted photons can be directly collected using a microspectral detection system, which is composed of an ANDOR detector (CCD-13448) and Omni-λ 500 spectrograph. Varying the injection current in the range of 0.5-9.4 mA, the obtained EL spectra are plotted in Fig. S4 (Support information), contributing to spontaneous emission. As the injection current reaches 11.6 mA, a sharp emission mode appearing at 390.5 nm can be seen, which is amplified due to optical oscillation in the MW microresonator. As the injection current increases above 11.6 mA, the corresponding EL spectra are plotted in Fig. 1c. Illustration in the figure, the EL spectra are governed by a group of sharp peaks in the ultraviolet band. More noticeably, no other oscillation peaks are found even at higher injection levels. There is little variations of the line shape and peak position of the EL spectra by increasing the injection current above 13.98 mA. The variations of the integrated EL intensity and spectral linewidth versus injection current are given in Fig. 1d. At low injection currents, the EL is dominated by broad spontaneous emission peaking at 389.0 nm and a spectral linewidth of ~31.5 nm. With an increase in the input current to 11.6 mA, a sharp peak with a full width at half maximum (FWHM) of ~0.18 nm suddenly emerges at 390.5 nm from the broad EL spectrum, which can be treated as a typical evolution from spontaneous emission to stimulated radiation^22^. Thus, a single optical mode was selectively amplified by the optical feedback in the hexagonal PtNPs@ZnO:Ga MW cavity. Additionally, the lasing threshold current is extracted to be ~11.12 mA, confirming the single-mode lasing operation upon electrical excitation. At an input current of 21.05 mA, the plotted EL spectrum is well fitted by a Lorentzian function^8,23^. As shown in Fig. 1e, the FWHM (*δλ*) is fitted to be 0.18 nm at a lasing wavelength (*λ*) of ~390.5 nm, indicating that the cavity quality *Q*-factor value is evaluated to about 2169 according to the formula *Q* = *δλ*·*λ*^−1^.

**Fig. 2:**
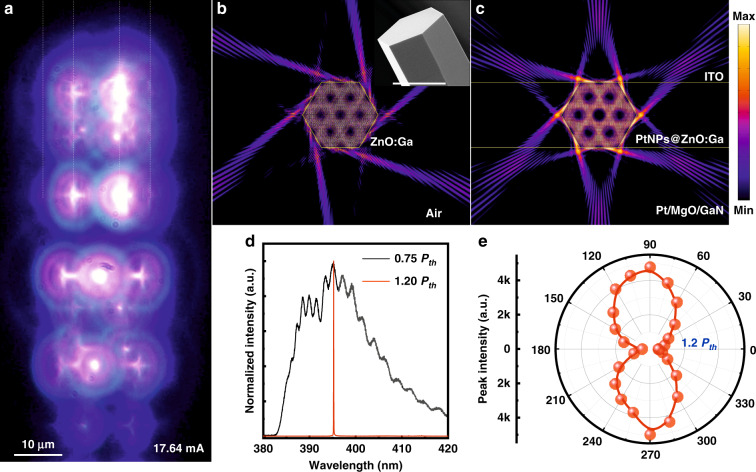
Device characteristics of the laser emission from the fabricated microlaser at the threshold. **a** Optical microscopic CCD image of the emission from the fabricated device. **b** Finite-element simulations showing the standing-wave electric-field pattern of a bare ZnO:Ga wire with hexagon-shaped cross section under an optical resonant mode. Inset: SEM observation of a bare ZnO:Ga wire, that showing the hexagon-shaped cross section (Scale bar: 15 μm). **c** The simulated resonant standing-wave electric-field pattern within the cross-section of the fabricated n-PtNPs@ZnO:Ga MW/Pt/MgO/p-GaN emission device structure. **d** Normalized EL emission spectra of the fabricated device for two injection currents below (0.75 *P*_*th*_), and above (1.20 *P*_*th*_) the laser threshold, respectively. **e** Polarization properties of the laser emission for an operating current above the threshold (~1.20 *P*_*th*_). The experiment EL data are symbolized by the red dots and the red solid line is the fitting result

As previously reported that, the individual ZnO:Ga wires with hexagon-shaped cross section can support WGM lasing, thus, the light can be well confined and propagated circularly in the cavity to form WGM resonance due to the multiple total internal-wall reflection^30,35^. To confirm the WGM lasing characteristics, Fig. 2a illustrates the CCD image of the recorded light-emission pattern at an operating current above the threshold (~17.64 mA). From the figure, the strongest lighting spots are distributed along the straight and sharp edges of the hexagon-shaped ZnO:Ga wire. Besides, a nonuniform light-emission intensity along the wire edges with several brighter lighting spots were also seen. The dark region observed in the light-emission regions along the wire edges could be derived from the nonuniform electronic contact at the interface. It’s worth noting that the bright emission can only be out-coupled from the sharp edges of the hexagon-shaped wire, suggesting the good optical confinement endowed by the light oscillation due to the total internal reflection at the boundary of ZnO:Ga wire cavity. More importantly, a given microlaser that can emit out the photons from the hexagonal ZnO:Ga MW WGM cavity and also the direction^35,36^. Using the finite-difference time-domain (FDTD) method, the light field confined within the hexagonal ZnO:Ga wire microcavity was simulated. In the simulation, the diameter of the ZnO:Ga MW is set to be 15 μm. The refractive indices of ZnO:Ga MWs, air, and Pt/MgO/GaN substrate are set to be 2.5, 1.0, and 2.0, respectively. The simulated result of a bare ZnO:Ga wire given in Fig. 2b has illustrated that the WGM lasing emission is predominantly emitted out from the 6 corners of hexagon, and the lasing intensity is periodically distributed around the hexagonal wire cavity^32,37^.

Enforcing the ZnO:Ga wire covered by PtNPs into the designed emission device, the cross-section was further simulated. Shown in Fig. 2c, the simulated light-field distribution reveals the optical field distribution of WGM resonance, as well as the output directions from the six corners of the hexagonal ZnO:Ga wire (Illustration in Fig. S5, Support information). To confirm the WGM lasing features of the fabricated single wire emission device, the EL spectra with normalized intensities were obtained at two injection currents below (0.75 *P*_*th*_) and above (1.20 *P*_*th*_) the threshold, and depicted in Fig. 2d for a comparison. It is significantly shown that the EL spectrum revealed a broad peak with the linewidth of about 31.5 nm, suggesting the spontaneous emission at low drive current. A series of sharp peaks are also acquired and its explanation can be resulted from the ZnO:Ga wire WGM cavity. Increasing the drive current above the threshold, the broad spectral line sharply collapsed into a single sharp peak with a dominant emission line at 390.5 nm. That is, the obtained EL spectrum is dominated by one bright and resolution-limited emission (The resolution of a spectrometer is limited to 0.1 nm), which can be assigned to the WGM cavity mode^18,34^. The polarization characteristics in the far-field light-emitting of the as-constructed MW emission device were further studied (see Materials and methods)^5,37^. By rotating the angle of the polarizer at the drive current of 17.64 mA, the emitted photons were recorded. Figure 2e reveals the variation of the laser emission intensity as a function of the polarizer angle, illustrating approximatively a cosine square variation. The degree of polarization was derived to about 0.85% according to the formula *P* = (*I*_*max*_ − *I*_*min*_)·(*I*_*max*_ + *I*_*min*_)^−1^, yielding an approximately linear polarization feature of the lasing emission. Taken the all into consideration, these observations supply compelling evidence for the achieving single-mode lasing emission from the as-designed single wire electrical-injection device structure at room temperature.

**Fig. 3:**
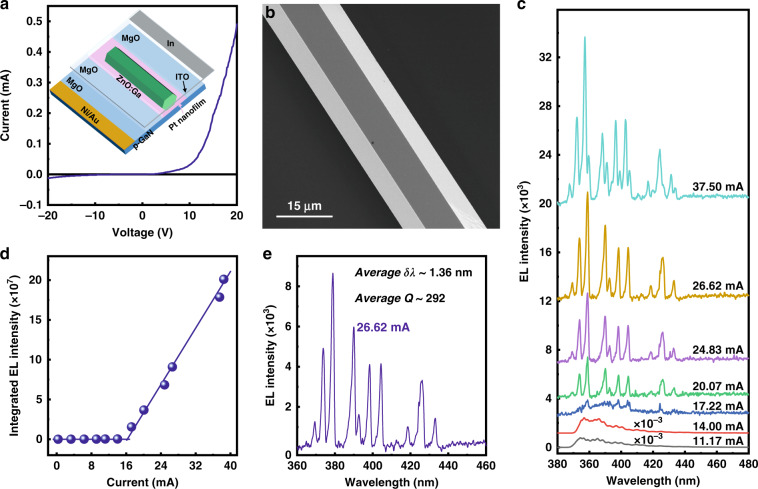
Fabrication and characterization of electrically driven microlaser, showing multimode lasing actions. **a** *I–V* curve of the fabricated n-ZnO:Ga MW/Pt/MgO/p-GaN heterojunction device. Inset: A schematic illustration of the fabricated ultraviolet microlaser, which is consisting of a bare ZnO:Ga MW, Pt/MgO buffer layers and p-type GaN film. **b** SEM observation of an individual ZnO:Ga MW, showing hexagon-shaped cross-sectional profile and smooth side surfaces. **c** Injection current-dependent EL emission spectra of the as-fabricated single MW heterojunction emission device, with the input current varying in the range of 11.17–37.50 mA. **d** Integrated EL intensity as a function of injection current, yielding a lasing threshold of 16.4 mA. **e** EL spectrum via Lorentz fitting at an operating current of 26.62 mA, providing the average linewidth of the lasing peak *δλ* ~ 1.36 nm, and the corresponding *Q*-factor is calculated to about 292

The diameter of the ZnO:Ga MW used in the as-constructed emission device is determined to be ~15 μm. This diameter is relatively larger than that of the optical wavelengths. Thus, the obtained single-mode lasing operation cannot be assigned to the reduction of the cavity length, which is related to the gain spectral bandwidth^8,12^. To research the single-mode operation, the influence of PtNPs on the lasing features should be examined. For a comparison, the same bare ZnO:Ga MW was utilized to construct the emission device (See Fig. S6 in Support information), and the device architecture based on the n-ZnO:Ga MW/Pt/MgO/p-GaN heterojunction is depicted in the inset of Fig. 3a. The electrical contact behavior was tested, and the *I–V* curve is shown in Fig. 3a, illustrating excellent rectification behavior. Therefore, a 1D wire heterojunction emission device was constructed. Figure 3b shows an SEM observation of the used ZnO:Ga MW, having a perfect hexagonal structure and smooth side facets. Upon forward bias, the EL characterization was also exploited to study emission characteristics of the n-ZnO:Ga MW/Pt/MgO/p-GaN heterojunction. Figure 3c gives the EL emission spectra at different injection current, illustrating multipeak light-emitting features with periods mainly distributed in the ultraviolet band. A dominant peak of the broad emission is centered at ~378.0 nm at low injection current, and the spectral linewidth was evaluated to about 20.0 nm.

When the drive current reaches 17.22 mA, several sharp peaks emerged over the broad emission band. Their intensities increase superlinearly with increasing the drive current. Varying the injection current in the range of 20.07-37.50 mA, the EL spectra are dominated by a series of sharp peaks. Thereby, as the injection current was increased, the transition to stimulated radiation and full lasing oscillation was observably achieved due to the sharp increase of spectral purity of the microresonator mode. The integrated EL peak intensity versus the injection current was depicted in Fig. 3d. Clearly, a typical two-stage input current-dependent emission relationship is observed, suggesting a distinct transition from spontaneous radiation to lasing emission. A threshold current of ~16.4 mA is derived. The lasing characteristics achieved in the fabricated single MW heterojunction microlaser was further studied. Figure 3e illustrates a sharp lasing peak extracted from Fig. 3c at the injection current of 22.62 mA. This peak can be fitted well with a Lorentzian function, yielding a narrow FWHM (*δλ*) of ~1.36 nm. Due to the formula of *Q* = *λ*·*δλ*^−1^, where *λ* and *δλ* are the center wavelength and FWHM of the peak, respectively. The corresponding *Q*-factor value is determined to be 292. In addition, the lasing feature is attributed to the random modes instead of the desired WGM order, which could be derived from the different reflective conditions at various side surfaces of the used ZnO:Ga wire with hexagon-shaped cross section, such as ZnO:Ga/air, ITO/ZnO:Ga, ZnO:Ga/Pt/MgO^18,24^. The present study shows that a bare ZnO:Ga MW with the diameter on the order of ten to dozens of microns cannot be utilized to achieve single-mode lasing in the as-fabricated heterojunction emission device.

**Fig. 4:**
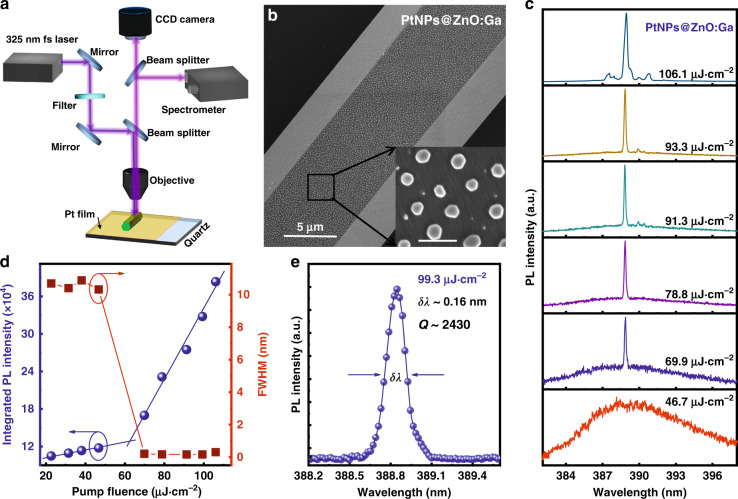
Lasing characterization of an individual PtNPs@ZnO:Ga MW upon optically pumped. **a** Schematic showing the home-built micro-PL measuring equipment, in which the single ZnO:Ga MW covered by large-size PtNPs was pumped by a 325 nm fs-amplified laser. **b** SEM image of a single ZnO:Ga MW covered by PtNPs. Inset: Enlarged SEM image of the PtNPs, which are deposited on the side surface of ZnO:Ga MW. The diameter of the deposited PtNPs is extracted to about 130 nm. **c** PL emission spectra of an individual PtNPs@ZnO:Ga MW at different pump power densities below and above the threshold. **d** Integrated PL intensity and spectral linewidth of the lasing peak as a function of pump fluence, yielding a threshold fluence of *P*_*th*_ ~ 66.2 μJ·cm^−2^. **e** Magnified PL spectrum of the 388.5 nm lasing mode. The dots and line are experimental data and Lorentz function fitting curve, respectively. The spectral FWHM of the lasing peak is extracted to about 0.16 nm, and the *Q*-factor value is calculated to ~2430

Lasing action achieved by the operation of optical pumping is the first and critical step to developing single-mode semiconductor laser devices. Thus, the lasing characterization obtained from optically pumped single ZnO:Ga MW not covered and covered by large-size PtNPs should be measured. Additionally, the incorporation of PtNPs on the lasing-mode manipulation and selection of cavity resonant modes was studied and may provide in-depth understanding of the material properties. In the experiment, the single-mode lasing operation achieved in the as-constructed n-PtNPs@ZnO:Ga MW/Pt/MgO/p-GaN heterojunction laser device can be ascribed to the introduction of PtNPs, which have been deposited on the wire. The effect of PtNPs on the mode management of a single ZnO:Ga MW microlaser was investigated at room temperature. Figure 4a schematically depicts the experimental setup of the home-built confocal μ-photoluminescence (PL) system using a pumping source of a 325 nm fs-amplified laser. In the lasing measurement, the laser beam was guided into the optical microscope objective to guarantee uniform excitation. The lasing characteristics of the ZnO:Ga wire not decorated and decorated by PtNPs are shown in the Supporting Information (Illustration in Figs. S7-S8 in Support information), indicating WGM lasing. The corresponding lasing threshold of about 91.5 μJ/cm^2^ is derived. After introducing PtNPs, lasing measurements were further performed under the same excitation condition. SEM image of a PtNPs@ZnO:Ga MW is shown in Fig. 4b, in which the average diameter of PtNPs is measured to about 130 nm. The PL spectra of the PtNPs@ZnO:Ga MW are shown in Fig. 4c. Illustration in the figure, the line shape reveals a clear transition from a much low-intensity broadband spectrum at low excitation levels to a dominant narrow peak at high excitation levels. As the pump fluence lower than ~46.7 μJ·cm^−2^, the PL spectra are dominated by broad spontaneous emission peaking at ~388.5 nm, and the FWHM is determined to be ~10.8 nm. As the pump fluence reaches 69.9 μJ·cm^−2^, the broad PL spectrum radically collapsed into a single sharp peak with a predominant luminescence line at ~388.8 nm, and a linewidth of ~0.16 nm is also obtained. By a comparison, the sharp peak emerged at the higher energy side of the multimode lasing spectra, which have been observed in the same bare ZnO:Ga MW. Further to increase pump fluence, the PL spectra are dominated by the sharp peaks. The PL peak intensity dramatically increases, and the peak position exhibits little shift. It indicates that the stable single-mode lasing action is achieved^8^. Figure 4d demonstrates the variation of integrated PL intensity and FWHM as functions of excitation power density for single-mode lasing. The slope of the output intensity versus the pump fluence can be observably fitted by two nonidentical linear parts, yielding a clear transition from spontaneous emission to lasing radiation. Therefore, the knee, named the lasing threshold, is evaluated to be *P*_*th*_ ~ 66.2 μJ·cm^−2^.

At a pump fluence of 99.3 μJ·cm^−2^, the PL spectrum is well fitted by a Lorentzian function (Fig. 4e). The FWHM of the lasing oscillation mode is extracted to about 0.16 nm, and the corresponding *Q*-factor is calculated to about 2430. The experimental results indicate that high-quality and excellent lasing emission with a single oscillation mode can be achieved in the as-prepared PtNPs@ZnO:Ga MW at room temperature. To test the validity, controllability and repeatability of achieving single-mode lasing, another single ZnO:Ga MW not covered, and covered by PtNPs, was pumped by pulse excitation. As illustrated in Fig. S9 in Support information, the bare ZnO:Ga MW exhibits WGM lasing characteristics via multiple modes. By introducing PtNPs (average diameter of the nanoparticles ~115 nm), the plotted PL spectra still exhibit multimode lasing characteristics. It is interesting to note that, the lasing peaks positioned at the lower energy shoulder are effectively suppressed. With an increase in the diameter of PtNPs up to 130 nm, only one sharp lasing peak at the high-energy side of the lasing spectrum is obtained. Significantly, we also find the mode number shows an observable reducing trend with increasing the diameter of the as-prepared PtNPs. However, as the size of PtNPs is further increased above 150 nm, the main absorption band would redshift to visible wavelengths, which is beyond the lasing band; while the optical loss will increase and the gain may not compensate the loss. The lasing action, as well as the related single-mode lasing operation is hard to occur in this case. Our results indicate that the incorporation of PtNPs with desired size can be utilized to lock a single lasing oscillation mode of ZnO:Ga MWs, rather than reducing the laser size to the subwavelength scale^8,12,14^.

**Fig. 5:**
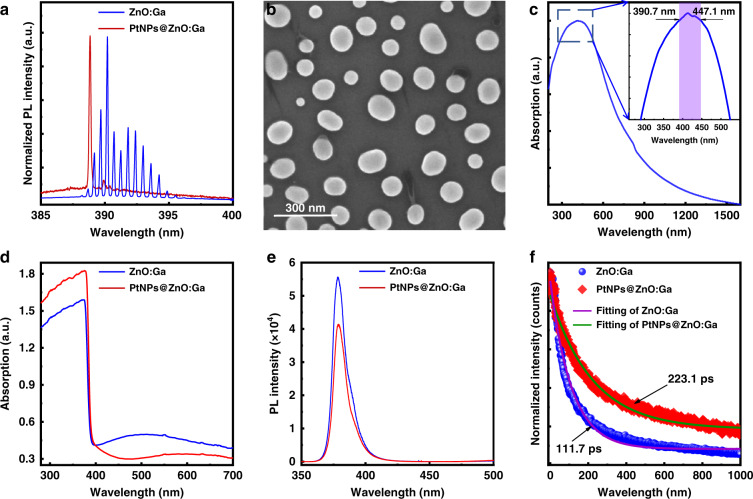
The influence of large size PtNPs on the optical properties and lasing features of a single ZnO:Ga MW. **a** Normalized lasing spectra of a single ZnO:Ga MW not decorated and decorated by PtNPs is given for a comparison. **b** SEM image of PtNPs prepared on a quartz substrate. **c** Normalized absorption spectrum of the as-prepared PtNPs. Inset: Enlarged absorption peak varying in the range of 390.7 ~ 447.1 nm. **d** Optical absorption spectra of a single ZnO:Ga MW and the same wire decorated by PtNPs. **e** PL spectra of a single ZnO:Ga MW not decorated and decorated by PtNPs. **f** Normalized TRPL spectra and exponential decay fitting curves at a wavelength of 390 nm of a single ZnO:Ga MW before and after PtNPs deposition

By comparing with the lasing features obtained in the bare ZnO:Ga MW, significantly mediated characteristics, containing reduced *Q*-factor, lower lasing threshold, and lower lasing intensity, can be concluded by incorporating the large-size PtNPs deposition. The corresponding working principle was studied. It could be found out that the realization of single-mode lasers cannot be assigned to the plasmonic response of PtNPs, which has been generated by the excitation of localized surface plasmon resonances^4,38^. This phenomenon is generally analogous to developing single-mode lasers induced by saturation absorption^39,40^. The optical properties of the prepared PtNPs deposited on the wires should be characterized. The normalized lasing spectra obtained from a single ZnO:Ga MW not decorated and decorated by PtNPs is shown in Fig. 5a for a comparison. This figure clearly illustrates that the obtained single-mode lasing peak of the PtNPs@ZnO:Ga MW is situated on the higher energy side of the PL spectrum, which has been obtained from that of the same bare MW. Accordingly, the rest of the lasing peaks of the bare wire are successfully suppressed by depositing PtNPs. PtNPs with identical size were prepared on a quartz substrate using the same experimental conditions. And the corresponding optical characterization was performed. As illustrated in the Fig. 5b, hemiellipsoid-shaped Pt nanoparticles are created due to surface tension and recrystallization, and the diameter of these nanoparticles is measured to ~130 nm. The optical properties of the as-prepared PtNPs were studied by ultraviolet-visible absorption spectroscopy. The corresponding absorption spectrum is plotted in Fig. 5c. The magnified main peak varying in the range of 390.7 ~ 447.1 nm was further shown in the inset of Fig. 5c. By comparing with our previously reported work, the achieved single-mode operation can be ascribed to the excellent absorption characteristics of PtNPs, instead of the plasmonic properties^41^.

The optical absorptions of a ZnO:Ga MW with/without PtNPs decoration were measured. The absorbance spectra shown in Fig. 5d demonstrate that the cutoff absorbance edges of both the samples are positioned at ~400 nm. More importantly, the absorption intensity of the ZnO:Ga MW is significantly increased by incorporating PtNPs, especially in the wavelength band of 200-400 nm. The ZnO:Ga MW not decorated and decorated by PtNPs, show much weaker absorption over the visible band. The cutoff absorbance edge of the wire covered by PtNPs shifts toward longer wavelength than that of the bare wire, accompanying by an enhanced absorption in the ultraviolet band^42^. In addition, PL spectrum of the single bare ZnO:Ga MW illustration in Fig. 5e (the blue solid line) exhibits a narrow and strong peak at 378.5 nm and an almost negligible broad emission in the visible band. The predominant ultraviolet luminescence can be regarded as the near-band-edge (NBE) emission of the ZnO:Ga MW, which has been extensively observed in various ZnO micro- and nanostructures. The broad visible emission may originate from intrinsic defects, and the possible Ga impurity-related doping levels in ZnO:Ga samples^43^. By introducing PtNPs, the obtained PL profile is much similar to that of the bare MW, accompanied by a noticeable decrease in emission intensity in the ultraviolet wavelengths (the red solid line, Fig. 5e). Obviously, the deposition of PtNPs cannot be employed either to enhance the NBE emission, or to suppress the broad visible luminescence.

For more insights into the microcavity regulation process of the PtNPs, time-resolved PL (TRPL) measurements for the ZnO:Ga MW with/without PtNP decoration were performed (Shown in Fig. S10, Support information). The TRPL results shown in Fig. 5f illustrate that the incorporation of large-size PtNPs reveals a longer decay time closing to 223.1 ps, which is larger than that of the bare ZnO:Ga MW (~111.7 ps). This reveals that the lasing processes of the single-frequency operation in the PtNPs@ZnO:Ga MW for energy transfer cannot be ascribed to the plasmonic mode by comparing with the photonic mode. And the results are significantly different from previously reported literature^44,45^. The ZnO:Ga MW covered by large-size PtNPs can feature as a microresonator and support single-mode lasing. Therefore, the achievement of single-mode lasing for the single PtNPs@ZnO:Ga MW can be assigned to the superabsorber behavior of the incorporated PtNPs instead of the plasmonic influence^39,41,46^.

**Fig. 6:**
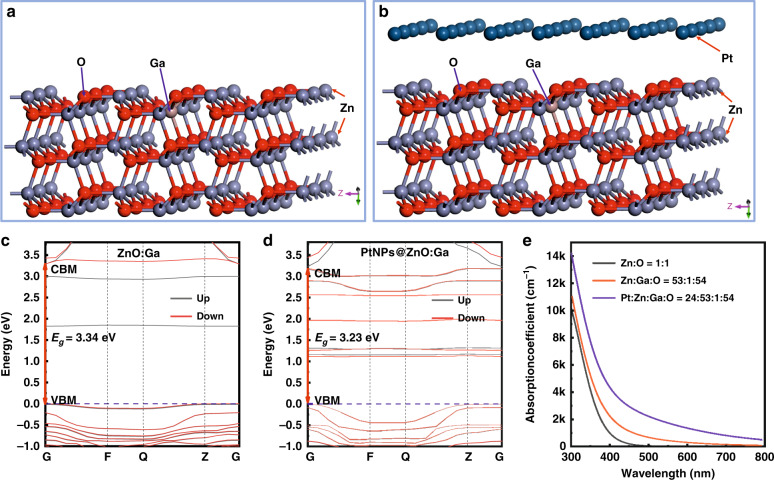
Theoretical analysis and calculation on the superabsorption characteristics of the large-size PtNPs. **a** Supercell structure of ZnO:Ga. **b** Structures of the PtNPs@ZnO:Ga interface: Zn termination (left) and O termination (right). **c** Band structure under spin polarization of ZnO:Ga system. **d** Band structure under spin polarization of ZnO:Ga covered by PtNPs. **e** Calculated optical absorption curves for ZnO, ZnO:Ga, and PtNPs@ZnO:Ga

The superabsorption properties of PtNPs were checked using first principles within density functional theory (DFT). Figure 6a, b demonstrate the supercell model diagram of Pt_24_Zn_53_Ga_1_O_54_, and Zn_53_Ga_1_O_54_, respectively^47,48^. The supercell models were built up on account of the pure ZnO system, which is obtained utilizing Materials Studio 8.0 software (See Fig. S11 in Support information). In the constructed supercell model, Pt atoms are adsorbed on the ZnO:Ga (1–1 0) surface. And a vacuum layer of 1.5 nm was inserted into the periodic boundaries between Pt atoms and ZnO:Ga along the z-axis direction. The band structures of the Pt_24_Zn_53_Ga_1_O_54_ and Zn_53_Ga_1_O_54_ supercells were calculated using the generalized gradient approximation (GGA) + U method. The calculated results are shown in Fig. 6c and d, respectively. The optical bandgap of the PtNPs@ZnO:Ga hybrid system (~3.23 eV) is narrower than that of the ZnO system doped with Ga element (~3.40 eV). The narrower bandgap suggests that electrons in the PtNPs@ZnO:Ga system can absorb lower energy photons, and then transition from the valence band to the conduction band. Accordingly, the generation of impurity levels is conducive to the staged transition of electrons in the system, thereby increasing the ultraviolet and visible light absorption in the PtNPs@ZnO:Ga system.

The calculated results of absorption profiles are shown in Fig. 6e. When Pt atoms are adsorbed on the ZnO:Ga surface, the optical absorption edge moves toward the lower energy side (a slight redshift). Hence, the optical absorption properties of PtNPs@ZnO:Ga MW hybrid systems located in the near-ultraviolet wavelengths, which is in accordance with experimental research, thus, the superabsorption can be established^44,49^. These results confirm that the realization of single-mode lasing operation can be attributed to the superabsorption characteristics of PtNPs, which have been prepared on the side surfaces of ZnO:Ga MW. These simulated results suggest that, the large-size PtNPs can heighten the absorption capability of the photons featuring in the near-ultraviolet wavelengths, thus breaking the original gain and loss. The experimental approach is possible that a number of competing resonator modes fall within the absorption bandwidth of the large-size PtNPs, which is inconsistent with the inherent active medium of the ZnO:Ga MWs. In this regime that within the superabsorption band of the large-size PtNPs, some of the modes experience either gain or loss, while the rest peaking in the higher energy side of the multimode emission band remains neutral^49–51^. We reveal that the incorporation of large-size PtNPs can be elegantly exploited to establish single-mode operation in inherently multimoded MW-microlasers, which being pumped optically and electrically. This is accomplished in a single ZnO:Ga wire structure covered by PtNPs with desired size, with one lasing-mode peaking at the higher energy side of multimode emission experiencing gain while the other lasing peaks provide an excess of loss.

## Discussion

In summary, a single-mode microlaser has been successfully constructed using a n-PtNPs@ZnO:Ga MW/Pt/MgO/p-GaN heterojunction. The proposed single-mode microlaser is electrically driven. The device exhibits excellent lasing performance with single-mode operation, consisting of a lasing peak at 390.5 nm, a narrow FWHM of ~0.18 nm, and a high *Q*-factor of ~2169. In the laser architecture, the inserted MgO nanofilm can function as a dielectric layer to engineer the band alignment of the ZnO:Ga/GaN heterojunction, lowing the electron leakage, yielding the electron-hole recombination in the single ZnO:Ga MW active media; while the insertion of Pt buffer nanolayer can facilitate the current injection and optimize the junction quality. Thus, the incorporation of Pt/MgO buffer nanofilms can integrate the depletion layer, laser medium, and microresonator in the single ZnO:Ga MW, realizing multimode lasing actions in the ultraviolet region. By manipulating the cavity resonant modes, the incorporation of PtNPs, potentially showing superabsorber behavior, can lock a desired lasing mode in the higher energy side of multimode lasing in the as-constructed single bare MW-based microlaser. The intriguing properties of PtNPs were also characterized experimentally and theoretically, illustrating that the lasing-mode selection and competition of a single ZnO:Ga MW can be modulated by incorporating PtNPs with desired sizes upon optically pumped. The achievement of single-mode microlasers with high repeatability and controllability was also examined. The experimental results provide an alternative strategy for the implementation of single-mode microlasers in the future.

## Materials and methods

### Device fabrication

Light-emitting devices composed of a single MW and p-GaN epitaxial plates were constructed. The fabrication procedure of the n-PtNPs@ZnO:Ga MW/Pt/MgO/p-GaN heterostructure device is summarized as follows: (i) In the device architecture, a p-type GaN film (commercially customized epitaxial plate) with a hole concentration of 4.05 × 10^18^ cm^3^ was selected. (ii) Ni/Au (35/50 nm) was deposited on the clean GaN layer using an electron-beam evaporation (EBE) system, serving as the p-type electrode. (iii) A MgO nanolayer with a thickness of ~8 nm (0.02 nm·s^−1^, 7.5 × 10^4^ Pa) was deposited on the p-GaN layer using the EBE technique. (iv) A Pt nanolayer with a thickness of 10 nm (0.02 nm·s^−1^, 7.5 × 10^4^ Pa) was further deposited on the MgO layer using the EBE technique. (v) A MgO film (0.1 nm·s^−1^, 7.5 × 10^4^ Pa) was prepared on the Pt film surface by using an antistatic mask blank. The thickness of the MgO film was about 2 μm. In the device, the prepared MgO films working as insulating layers can be used to prevent the direct contact between the top electrode (ITO) and p-GaN layer. Finally, an individual MW was transferred onto the Pt nanolayer. ITO, used as the n-type conductive layer, was placed on top of the MW. A schematic depiction of the as-fabricated 1D wire-type heterostructured light-emitting device is given in Fig. 1a.

### Device characterization

Using a Keysight semiconductor device analyzer (B1500A) as current source, the current-voltage properties of the fabricated device were measured. Upon electrical excitation, the emitted light was captured using a spectrometer and CCD detector. The optical microscopic images were recorded using a high-definition color CCD camera above the top-surface of the as-fabricated emission device. The polarization properties was resolved by rotating the rotatory polarizer (Glan-Taylor calcite) through the microscope objective with a high numerical aperture, and the polarizer was installed on the top-surface of the device.

### Theoretical models and calculation method

The calculations were performed using the first-principles pseudopotential method based on DFT within the Cambridge Sequential Total Energy Package (CASTEP) as implemented in Material Studios 8.0^47,48,52^. The GGA + U method of Perdew-Burke-Ernzerhof (PBE) was applied to describe the exchange-correlation function. Vanderbilt-form ultrasoft pseudopotentials with a valence electron configuration of *3d*^*10*^*4s*^*2*^ for Zn, *2s*^*2*^*2p*^*4*^ for O element, *3d*^*10*^*4S*^*2*^*4p*^*1*^ for Ga element, and *5d*^*9*^*6s*^*1*^ for Pt element were used to depict the interaction between the valence electrons and ionic core. The plane-wave basis set was used to expand the electron wave functions, and the cutoff energy was selected as 300 eV. Other computational details are displayed in Table S2. All structural parameters for ZnO were close to the experimental values and other calculations^44,52^.

## Supplementary information

Supplementary information for Electrically driven single microwire-based single-mode microlaser
