# Supplementary material for: Electrically driven single microwire-based single-mode microlaser
Source: Light Sci Appl. 2022 Jun 29;11:198. doi: 10.1038/s41377-022-00874-w (PMC9240044; doi:10.1038/s41377-022-00874-w)
Supplement: Supplementary file 1 — Supplementary information for Electrically driven single microwire-based single-mode microlaser [file 41377_2022_874_MOESM1_ESM.docx]

Supplementary information for Electrically driven single microwire-based single-mode microlaser

Xiangbo Zhou, Mingming Jiang,* Kai Xu, Maosheng Liu, Shulin Sha, Shuiyan Cao, Caixia Kan,* and Da Ning Shi*

Correspondence author:

Mingming Jiang (Email: [mmjiang@nuaa.edu.cn](mailto:mmjiang@nuaa.edu.cn); Telephone number: 18851870557) ,

Caixia Kan (Email: [cxkan@nuaa.edu.cn](mailto:cxkan@nuaa.edu.cn);Telephone number: 13770798225),

and Da Ning Shi (Email: [shi@nuaa.edu.cn](mailto:shi@nuaa.edu.cn); Telephone number: 13851890589)

^a^ College of Physics, MIIT Key Laboratory of Aerospace Information Materials and Physics, Key Laboratory for Intelligent Nano Materials and Devices, Nanjing University of Aeronautics and Astronautics, No. 29 Jiangjun Road, Nanjing 211106, China.

**Supplementary Section S1 | Sample characterization of a single ZnO:Ga MW.**

As we previously reported, ultralong ZnO:Ga MWs with straight sidewalls, perfect hexagonal morphology and fine crystallinity, were successfully fabricated through a simply reproducible CVD method in horizontal tube furnace^1^. Optical photograph of the synthesized samples is illustrated in Fig. S1a and the synthesized individual MWs have widths of several microns to several dozen microns, and lengths of several hundred millimeters to two or three centimeters. These MWs appear very straight and stiff despite their long lengths. Phase structure of the as-synthesized ZnO:Ga MWs was studied by using Micro-region X-ray diffraction (Micro-XRD). Micro-XRD pattern illustration in Fig. S1b, demonstrates strong diffraction peaks, which can be assigned to the wurtzite structural ZnO with good crystal quality. High-resolution transmission electron microscopy (HRTEM) image of a ZnO:Ga MW with a straight boundary was characterized, and the corresponding HRTEM image was shown in Fig. S1c. From the figure, the wire demonstrates clear lattice fringes, and the interplanar distance is extracted to be about 0.275 nm, which is larger than that of intrinsic ZnO (The interplanar distance of intrinsic ZnO is about 0.260 nm)^2^. It suggests that the lattice expansion of a ZnO MW incorporated by Ga-element can be attributed to the substitution of Ga for Zn.

**
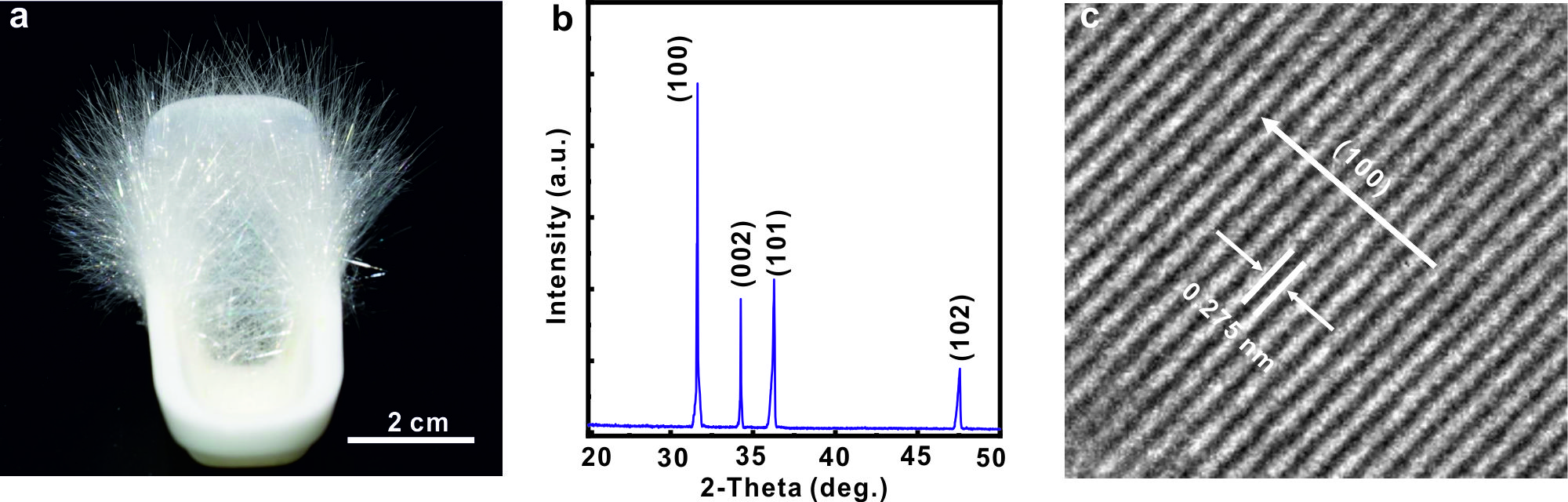
**

**Fig. S1** **a** Optical photograph of an individual ZnO:Ga MWs. **b** Micro-XRD peaks of an individual ZnO:Ga MWs. **c** High-resolution TEM image of a single ZnO:Ga MW.

**Supplementary Section S2 | Preparation of physically isolated PtNPs.**

The incorporation of Ga-doping can realize tunable n-type conduction of individual ZnO MWs. Especially, due to the outstanding electronic transport properties, an individual ZnO:Ga MWs can be employed to construct semiconducting incandescent-type emitter^3^. Taken a single ZnO:Ga MW into account, incandescent-type emitter was fabricated. As the applied bias reached a certain value, bright and green light emission can be observed, the corresponding optical picture is shown in Fig. S2a. By introducing Pt film (the sputtering time 600 s), the EL features of the bare MW was modulated, the optical image of light emission is captured. Detailed information on the red illuminating from electrically driven incandescent-type emission device, composed of single ZnO:Ga MW covered by Pt nanofilms, was further shown in Fig. S2b. To study the influence of the Pt nanostructures on the luminescence features of a single ZnO:Ga MW, surface morphology of the wire was characterized. Fig. S2c demonstrates typical SEM image of a bare ZnO:Ga MW, illustrating smooth surfaces. By incorporating Pt nanofilms, the segment of the wire located in the lighting zone was characterized, and the corresponding SEM image is depicted in Fig. S2d. Clearly, PtNPs with physically isolated and relatively uniform size, can be prepared on the surfaces of a ZnO:Ga MW. An enlarged SEM of PtNPs@ZnO:Ga MW is depicted in Fig. S2e. Accordingly, the size and gap distance of PtNPs can also be modulated by varying the evaporation time. Further to confirm the preparation of PtNPs on the ZnO:Ga MW, the corresponding energy dispersive X-ray spectroscopy (EDS) mapping of PtNPs@ZnO:Ga MW illustrates a uniform spatial distribution of Ga, Pt, Zn and O elements, as illustrated in Fig. S2f-i. It is notable that, PtNPs can be prepared on the ZnO:Ga MWs, yielding uniform distribution. Therefore, an individual ZnO:Ga MW covered with PtNPs (PtNPs@ZnO:Ga) was successfully fabricated.


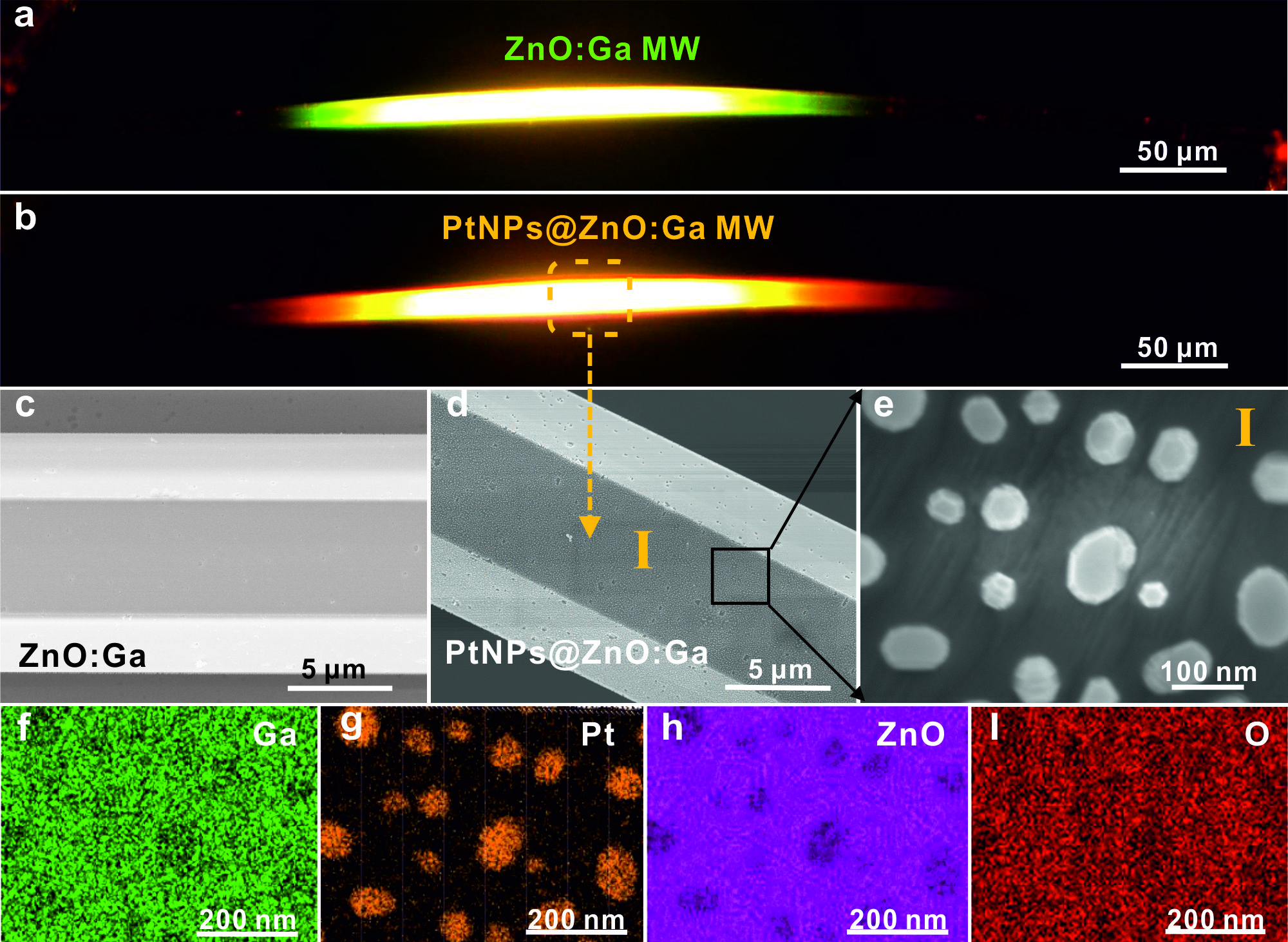


**Fig. S2** **a** Optical microscopy image of the bright green light emission from the electrically illuminated single ZnO:Ga MW-based incandescent-type light source. **b** Optical microscopy image of the bright red emission from the electrically lit up single MW based incandescent-type emitter, with the wire covered by Pt nanoparticles. **c** SEM image of a ZnO:Ga MW. **d** SEM image of an individual ZnO:Ga MW, which covered by physically isolated PtNPs. **e** SEM image of PtNPs, which prepared on the ZnO:Ga MW. **f-i** The corresponding EDS mapping showing the uniform distribution of Ga, Pt, Zn and O elements.

**Supplementary Section S3 | Device Fabrication.**

Electrically driven luminescence devices composed of single PtNPs@ZnO:Ga MW, and p-GaN epitaxial plates serving as hole injecting layer, were designed. The fabricating procedure of n-PtNPs@ZnO:Ga MW/p-GaN heterostructure device was summarized as follows: (i) In the device architecture, p-type GaN film (Commercially customized epitaxial plates) was selected with the hole concentration of 4.05×10^18^ cm^3^; (ii) Ni/Au (35/50 nm) was deposited on the GaN layer via the electron-beam evaporation system, serving as the anode. (iii) A MgO nanofilm via the thickness 8 nm (0.02 nm·s^-1^, 7.5×10^4^ Pa) was deposited on the p-GaN layer using the electron-beam evaporation technique; (iv) Afterwards, a Pt nanofilm with the thickness of 10 nm (0.02 nm·s^-1^, 7.5×10^4^ Pa) was deposited on the MgO layer using the electron-beam evaporation technique. (v) A rectangular hole via MgO working as dielectric layer (0.1 nm·s^-1^, 7.5×10^4^ Pa) was prepared on Pt film surface by using antistatic mask blank. The depth of rectangular hole was about 2 μm. In the device, the prepared MgO film working as an insulating layer can be used to avoid contact between the top electrode and p-GaN layer.

Finally, an individual MW was transferred to the rectangular micro-hole. ITO using as the cathode conductive layer was placed on the MW. Schematic description of the fabricated one-dimensional heterostructured luminescence device, was schematically illustrated in Fig. S3.


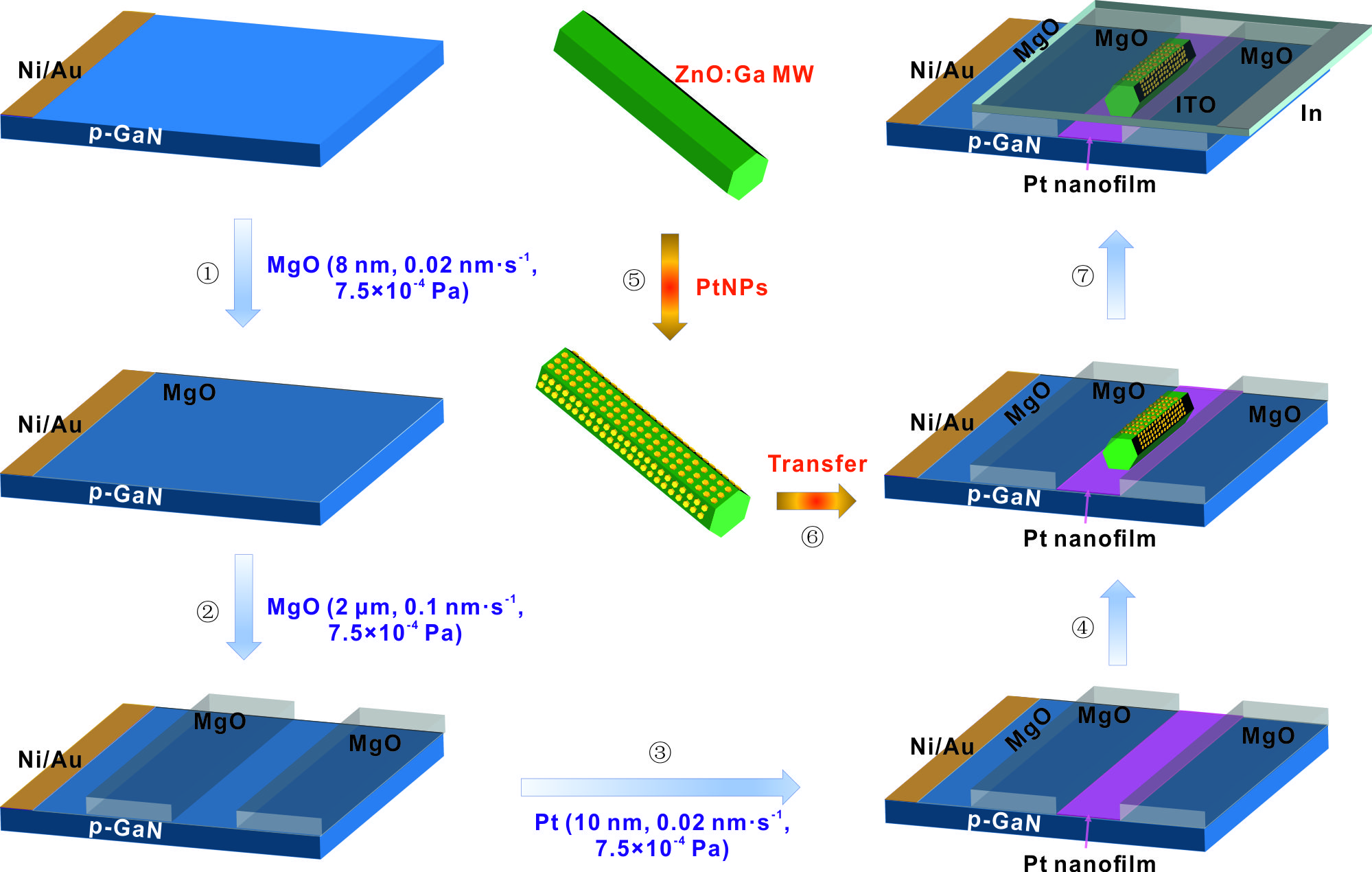


**Fig. S3** Schematic diagrams of key fabrication processes for electrically driven light-emitting device, made of an individual ZnO:Ga MW covered by PtNPs and p-type GaN substrate.

**Table S1 |** **Structure parameters of p-GaN layer.**

| **GaN** | **parameters** |
| --- | --- |
| Growth substrate | PSS single 430+/-25um |
| Growth direction | Grow along the (0001) direction (C plane) |
| Undoped GaN layer thickness | 3.5 μm |
| p-GaN layer thickness | 2.5 μm |
| p-type layer doping element | Mg (30%) |
| Carrier Density | 4.05×10^18^ cm^-3^ |

**Supplementary Section S4 | EL characterization of the as-constructed single n-PtNPs@ZnO:Ga MW/Pt/MgO/p-GaN light-emitting devices.**


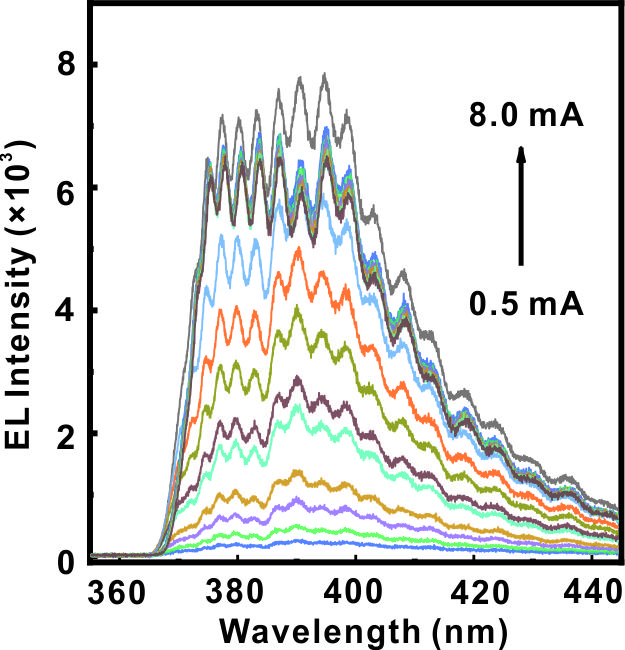


**Fig. S4**  EL spectra of the single n-PtNPs@ZnO:Ga MW/Pt/MgO/p-GaN heterojunction diode with forward injection currents range from 0.75 mA to 9.4 mA.

**Supplementary Section S5 |** **Optical microscopic CCD image of the emission from the n-PtNPs@ZnO:Ga MW/Pt/MgO/p-GaN light-emitting devices.**

**
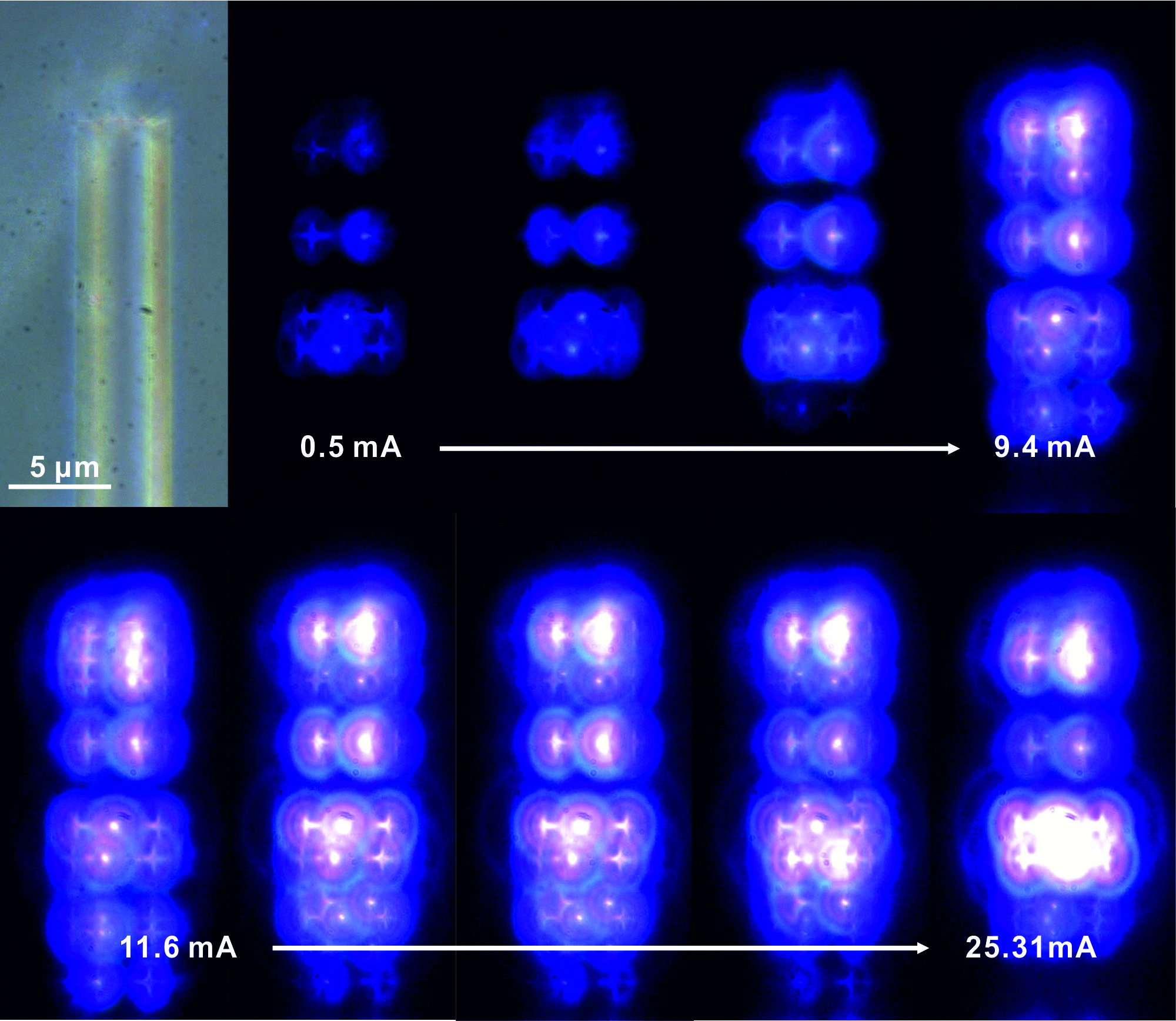
**

**Fig. S5**  Optical microscopic CCD image of the emission from the fabricated device.

**Supplementary Section S6 |** **EL characterization of the as-constructed single n-ZnO:Ga MW/MgO/p-GaN light-emitting devices.**

The schematic illustration of p-GaN/MgO/ZnO:Ga heterojunction is shown in Fig. S6a. Electrical characterization of the fabricated device was tested. The I-V curve illustration in Fig. S6b, indicates typical rectifying behavior, yielding p-n junction between n-ZnO:Ga MW and p-GaN film^4^. The turn-on voltage is estimated to be about 5.0 V. Fig. S6c displays the EL spectra of the heterostructured device by varying injection currents in the range of 0.47-11.31 mA. A broad ultraviolet emission was observed, with the main wavelengths emitting at around 380.5 nm. The integrated EL intensity was calculated as a function of the injection current, as shown in Fig. S6d. It exhibits a super-linear tendency, indicating the co-existence of radiative and nonradiative processes. In the device architecture, the incorporation of MgO layer between GaN and ZnO:Ga could block the electrons entering the GaN film, leading to engineering the band alignment^5^.


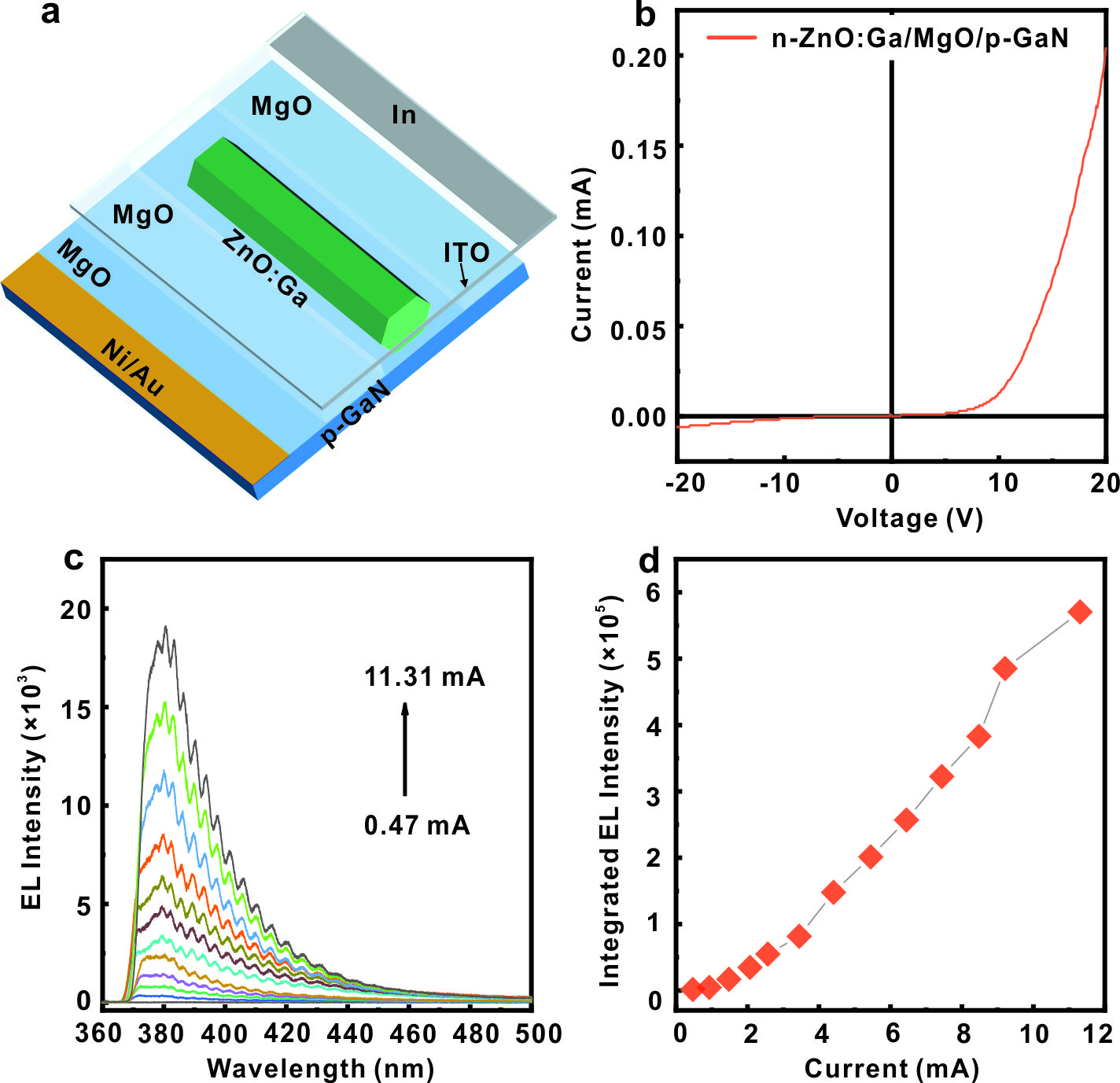


**Fig. S6** **a** Schematic diagram of the n-ZnO MW/MgO/p-GaN heterojunction device structure. **b** I-V curve illustrated LED-like rectifying characteristic, suggesting the formation of a p-n junction between ZnO:Ga MW and p-GaN layer. **c** EL spectra of the single ZnO:Ga MW based heterojunction LED with forward injection current ranging from 0.47 to 11.31 mA. **d** Integrated EL intensity as a function of the injection current of the fabricated heterojunction LED.

**Supplementary Section S7 | Lasing behavior of a single ZnO:Ga MW**

It is well-known that, the as-synthesized hexagonal MWs with the smooth side surfaces can potentially serve as the reflecting mirrors for fabricating typical Whispering Gallery Mode (WGM) cavities^6^. Together with the excellent optical gain character of a single ZnO:Ga MW, it is highly possible to achieve WGM lasing from an individual MWs. Schematic of the optically pumped a single ZnO:Ga MW based WGM microlaser is depicted in Fig. S7a. Optical characterization of WGM lasing is demonstrated, with a single MW is pumped optically by using a fs pulsed laser (excitation wavelength: 325 nm; repetition rate: 1 kHz; pulse length: 100 fs) composed of an optical parametric amplifier (OPERA SOLO) with a Ti:sapphire laser (coherent) and a confocal micro-PL system (Olympus BX53). The optical spectra were recorded by a spectrometer (SpectraPro-2500i, Acton Research Corporation). As individual ZnO:Ga MWs with hexagonal cross section naturally form a WGM microresonator, with six-lateral sides functioning as reflecting mirrors. To confirm the self-formed WGM cavity, numerical simulation of the corresponding resonant modes with eigenfrequency study in the hexagon-shaped cross section of the wire was performed by using finite difference time domain (FDTD) method^7^. During the simulation, perfect matched layer (PML) was utilized to absorb the outgoing waves. In a modeling, a single ZnO MW with hexagonal cross-section placed on a quartz substrate was constructed. The diameter of the MW (*D* = 10 mm), the calculated wavelength *λ* = 390 nm; the corresponding refractive indices of ZnO *n_ZnO:Ga_* = 2.35, quartz substrate *n_quartz_* = 1.5, and *n_air_* = 1.0, respectively. As shown in Fig. S7b, WGM resonant oscillation in the hexagon-shaped cross section of a single ZnO:Ga MW was well simulated numerically.

Fig. S7c shows the pumping-fluence dependent PL spectra of a single ZnO:Ga MW. A broadband spontaneous radiation with a spectral linewidth of about 15.0 nm can be observed around 391.0 nm when the pumping fluence is less than 69.5 μJ·cm^-2^. As the gradual increase in excitation fluence to 99.3 μJ·cm^-2^, some evenly spaced sharp spikes can be captured in the spontaneous emission spectrum; meanwhile the light intensity over the PL spectra versus the pumping-fluence illustrates a nonlinear increase feature, indicating that optical microresonanator is formed in the hexagonal cross-section of the MW. Generally, lasing action from optically pumped a hexagonal ZnO MW is dominantly assigned to WGM.^6^ Fig. S7d demonstrates a PL spectrum at the pump fluence of 122.2 μJ·cm^-2^. From the figure, uniformly spaced oscillation peaks (*Δ*λ ~ 0.56 nm), and full width at half maximum (FWHM) (*δ*λ ~ 0.11 nm) can be obtained, suggesting that the same waveguide origin of the optical modes. The average *Q*-factor was estimated as 3317 according to the formula *Q* = λ·*δ*λ^-1^, where λ is the peak wavelength.^8^ The integrated light intensity (blue solid square) and spectral FHWM (red solid square) as function of the pumping power density are plotted in Fig. S7e. The fitted result of the integrated light intensity data clearly exhibits nonlinear behavior for lasing oscillation; While, the spectral FWHM sharply decreases from 15.0 to 0.11 nm. From the curves, the threshold pumping power density (*P_th_*) is determined to be about 91.5 μJ·cm^-2^. According to the experimental results, the luminescence intensity increases slowly as the excitation density is lower than *P_th_*, and the luminescence intensity dramatically increases when the pumping density is higher than *P_th_*. Meanwhile, the FWHM decreases from 15.0 nm to 0.11 nm as well, which depicts the transition from the spontaneous emission to the stimulated emission^5^.


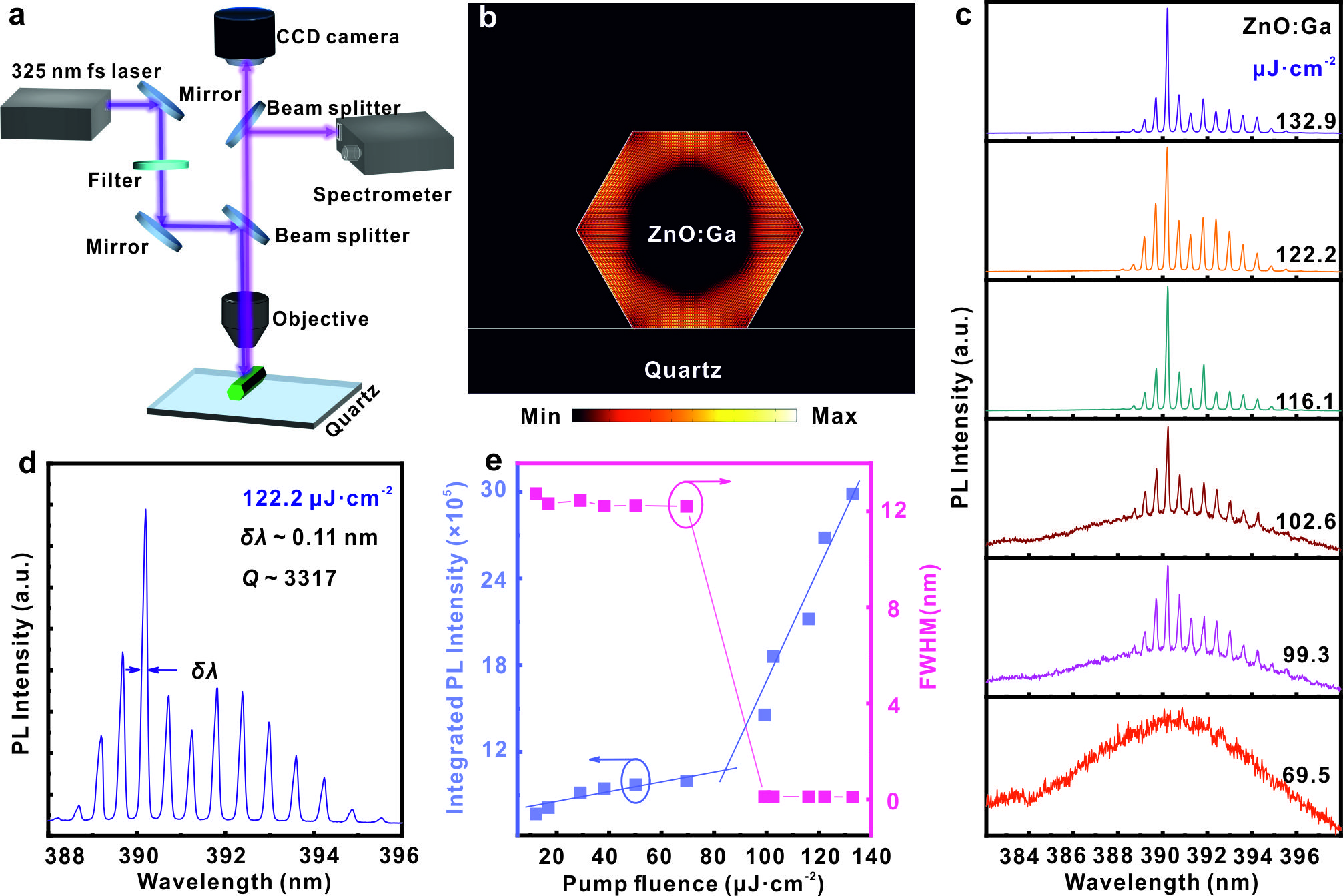


**Fig. S7**  **a** Schematic of an individual ZnO:Ga MWs placed on quartz substrate. The single MW is pumped optically by using a fs pulse laser (the pumping wavelength: 325 nm, repetition rate: 1 kHz; pulse length: 100 fs). **b** Simulated electric-field intensity |E|^2^ pattern of a single ZnO:Ga MW with hexagonal cross section, showing the well-supported whispering-gallery mode. The field distributions correspond to the fundamental mode. In the simulation, the incident wavelength λ = 385 nm, the diameter of the wire is *D* = 10 μm. **c** Pump fluence-dependent PL spectra at different pump power density ranging from 69.5 to 132.9 μJ·cm^-2^. **d** Typical lasing spectroscopy of the bare ZnO:Ga MW at the pump power density of 122.2 μJ·cm^-2^. **e** Integrated PL intensity and linewidth of the lasing peak as a function of pump fluence, indicating a threshold fluence of 91.5 μJ·cm^-2^.

**Supplementary Section S8 | Lasing mode number analysis of a single ZnO:Ga MW.**

To analyze the PL emission feature in more details, a calculation of the optical microcavity was performed. In a WGM microcavity, the resonant wavelength λ and the corresponding mode number ~N can be deduced as^6^

$N=\frac{3\sqrt{3}nD}{2\lambda}- \frac{\pi}{6}\tan^{-1} \left( 3\sqrt{{3n}^{2}-4} \right)$ (1)

where n is the refractive index of ZnO:Ga, and D is the diameter of the MW. Consider that, the transverse electric (TE) polarization light can be described using the Sellmeier's dispersion function^6^

$n\left( \lambda\right)=\left( 1+ \frac{{2.4885\lambda}^{2}}{\lambda^{2}-{102.3}^{2}}+\frac{{0.215\lambda}^{2}}{\lambda^{2}-{372.6}^{2}}+\frac{{0.255\lambda}^{2}}{\lambda^{2}-{1850}^{2}} \right)^{\frac{1}{2}}$ （2）

Accordingly, an optical mode calculation of the PL spectrum was performed. The theoretical modes was in great consistence with the experimental PL emission, which is further shown in Fig. S8. It can be inferred that lasing action from a single ZnO:Ga MW belonged to WGM, wherein the incident light is completely reflected with respect to the normal of the six lateral facets of MW. Similarly, the as-synthesized a single ZnO:Ga MW with hexagon-shaped cross section can give a higher *Q*-factor, thus, the smooth surfaces of the MW can support total reflection in all the side planes.  All the results indicate that WGM lasing can be obtained on account of a ZnO:Ga MW with hexagonal cross-section, in which the sidewalls of the MWs served as the reflecting mirrors of the optical concentrator that caused the total internal reflection^3^. Therefore, a single ZnO:Ga MW with hexagonal cross-section can be utilized to construct WGM microlaser.


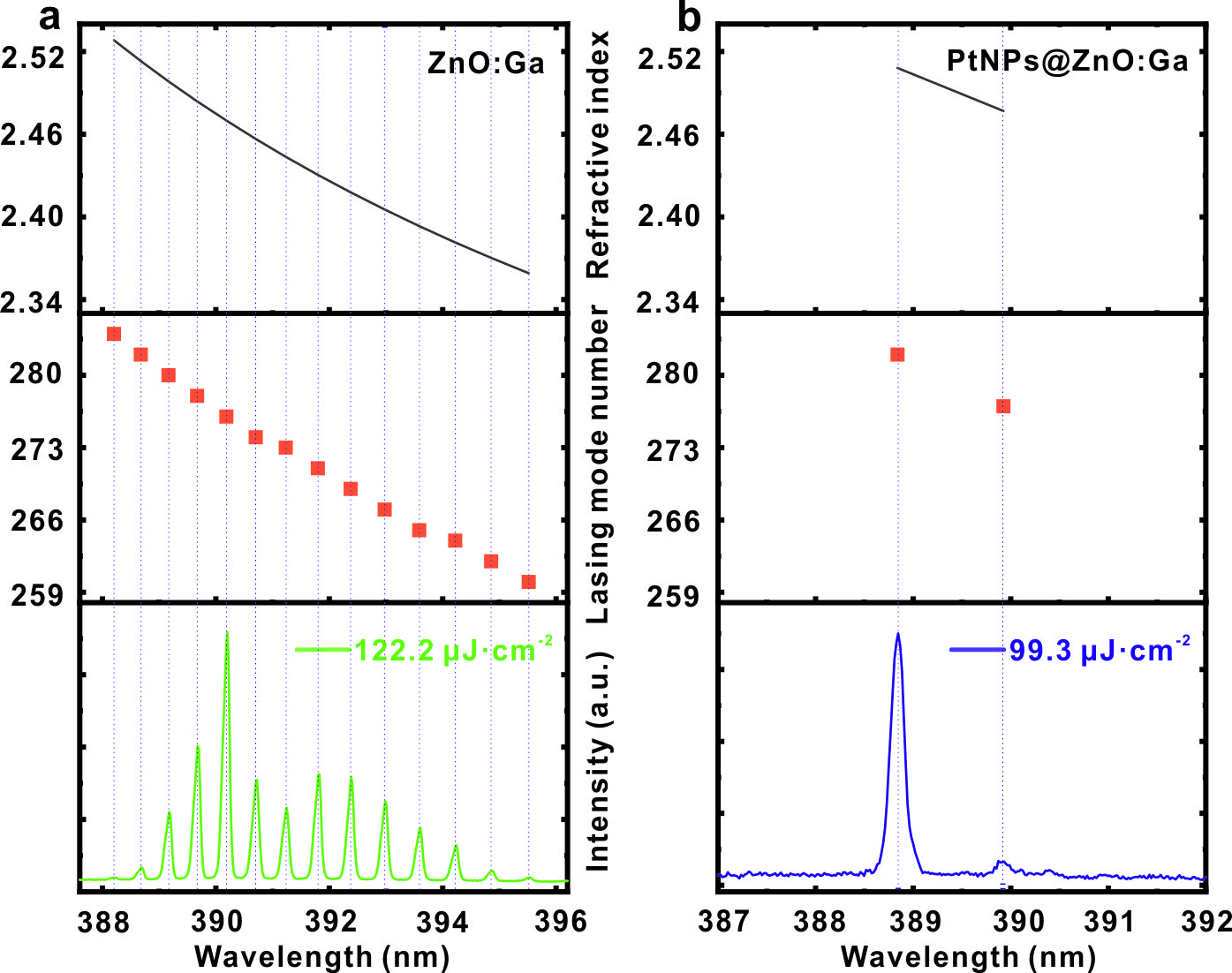


**Fig. S8**  **a** Lasing spectrum of a single ZnO:Ga MW at excition power density of 122 μJ·cm^-2^, wavelength-dependent lasing mode number *N(λ)* (red solid cube) and the dispersion relation of effective refractive index n(λ) versus λ (black line), respectively. **b** Lasing spectra of the wire covered by PtNPs (the excition power density of 99.3 μJ·cm^-2^), the calculated wavelength-dependent lasing mode number *N(λ)* (blue solid cube) and the dispersion relation of effective refractive index n(λ) versus λ (black line), respectively.

**Supplementary Section S9 | Modulation of the lasing features of the ZnO:Ga MW by PtNPs.**

Fig. S9a shows the excitation power density dependent PL spectra of the single bare ZnO:Ga MW. At a low excitation power density of 64.1 μJ·cm^-2^, the PL spectrum presents a broad spontaneous emission, which comes from the near band-edge (NBE) emission of ZnO:Ga. When the excitation power reached 67.6 μJ·cm^-2^, some sharp peaks with identical mode spacing appeared in the spectrum, indicating the occurrence of lasing action. With the incorporation of PtNPs (The size of the Pt nanoparticles is 115 nm), the same MW was also optically pumped by using a fs-pulsed laser. Fig. S9b shows the PL spectra as a function of the excitation power density. As the pump fluence increased above 24.8 μJ·cm^-2^, lasing action can also be achieved from the wire covered by PtNPs. It is worth noting that the lasing peaks at the lower energy side of the PL spectra are suppressed. As the size of the PtNPs increased up to about 130 nm (Fig. S9c), a single luminescence peak via narrower mode emits at around 385.8 nm, and then governs the PL spectra by varying the pump fluence. The corresponding FWHM sharply narrows to about 0.26 nm. It indicates that the implementation of single-mode lasing action is achieved.


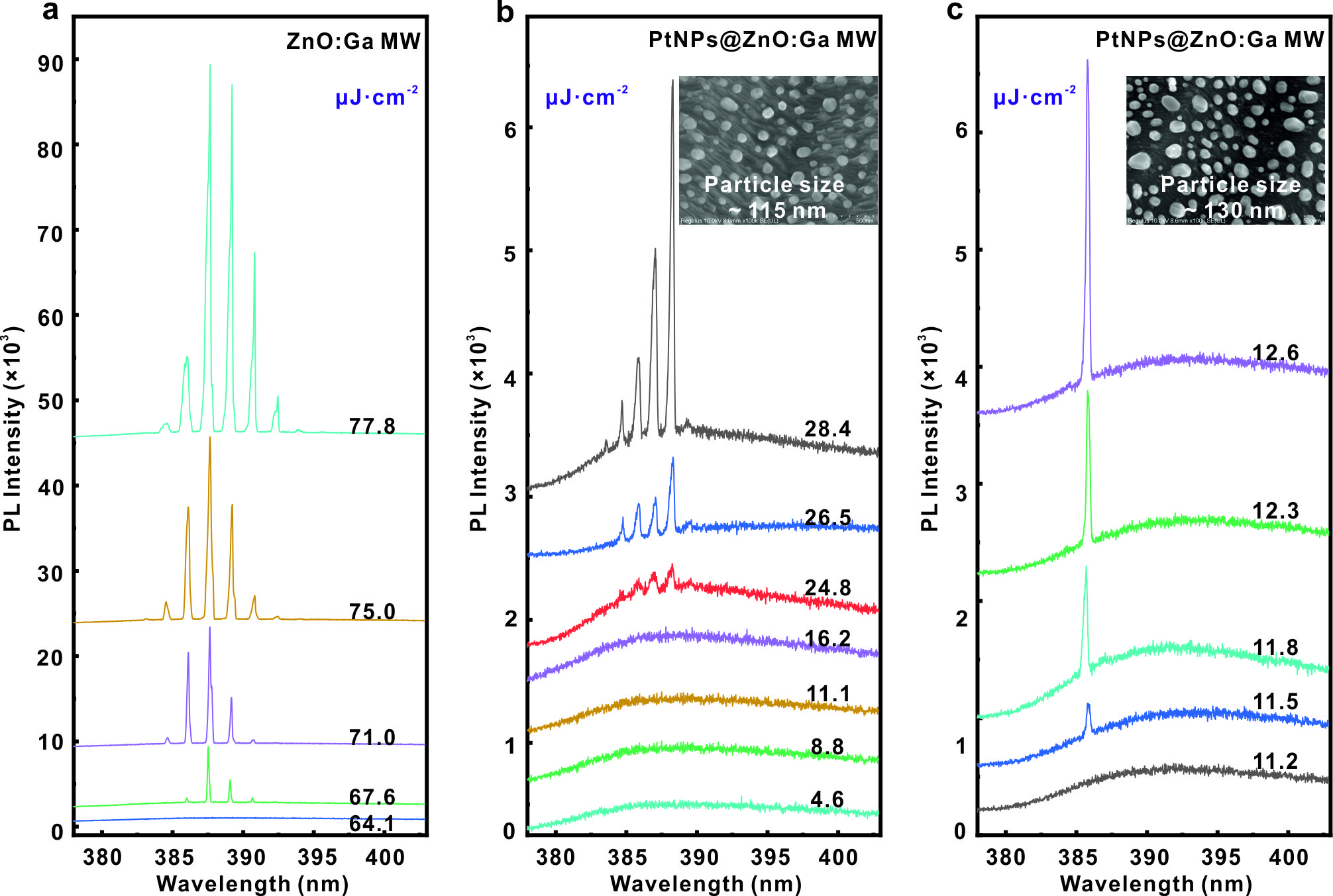


**Fig. S9 a** Pump fluence-dependent PL spectra of a single ZnO:Ga MW, with the pump power density ranging from 64.1 to 77.8 μJ·cm^-2^. **b** Pump fluence-dependent PL spectra of a single PtNPs@ZnO:Ga MW (The size of the Pt nanoparticles is 115 nm), with the pump power density ranging from 4.6 to 28.4 μJ·cm^-2^. **c** Pump fluence-dependent PL spectra of a single PtNPs@ZnO:Ga MW (The size of the Pt nanoparticles is 130 nm), with the pump power density ranging from 11.2 to 12.6 μJ·cm^-2^.

**Supplementary Section S10 | TRPL characteristics of a single ZnO:Ga MW with and without PtNPs.**

For more insights into the microcavity regulation process of the PtNPs, time-resolved PL (TRPL) measurements for the ZnO:Ga MW with/without PtNP decoration were performed. The temporal spectroscopic profiles collected using a streak camera are displayed in Fig. S10. The normalized TRPL decay curves of the wire can be well fitted using a monoexponential function. The decay lifetime is defined as follows^8^:

*I(t) = I_0_ e^(-t/τ)^,*  (3)

where *I_0_* and *I* are the fitting parameters. As shown in Fig. 5f, the calculated decay times are extracted to be approximately 111.7 ps and 223.1 ps for the bare ZnO:Ga and PtNPs@ZnO:Ga MW, respectively. Clearly, the incorporation of PtNPs cannot accelerate the recombination of carriers in the ZnO:Ga MW. Therefore, the achievement of single-mode lasing for the single PtNPs@ZnO:Ga MW can be assigned to the superabsorber behavior of the incorporated PtNPs instead of to a plasmonic influence^9,10^.


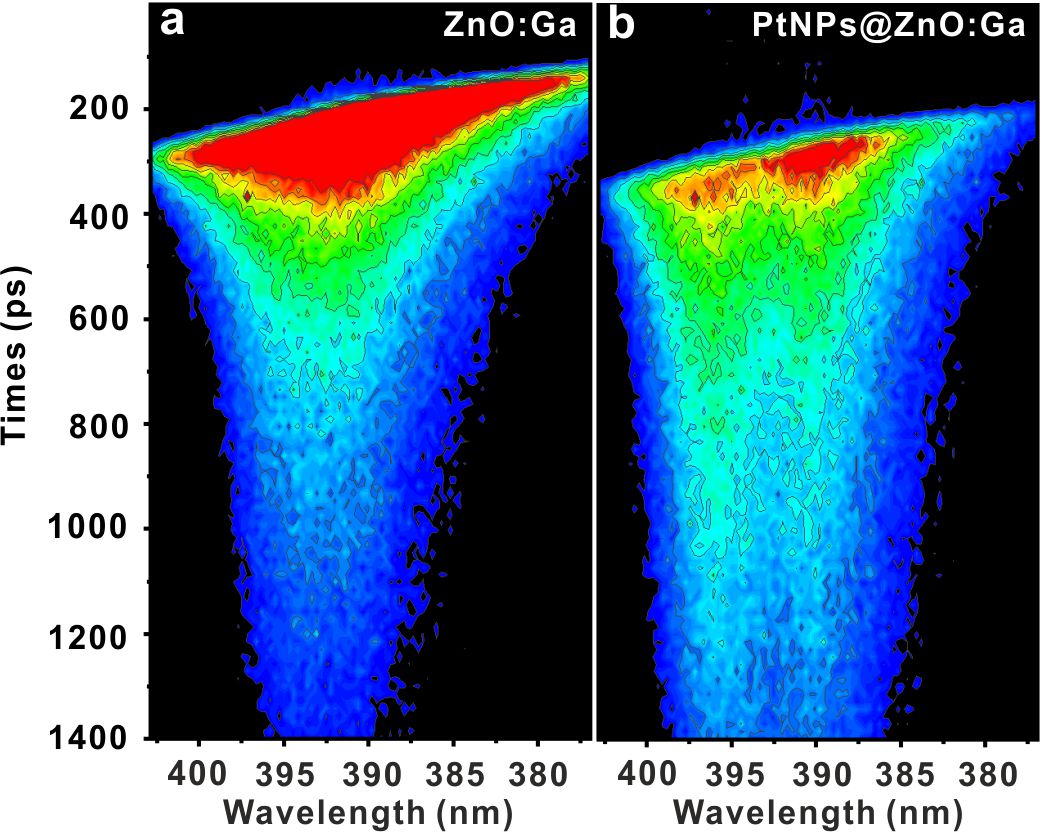


**Fig. S10** Temporal spectroscopic profile of **a** ZnO:Ga and b PtNPs@ZnO:Ga samples excited by 355 nm laser and collected by a streak camera.

**Supplementary Section S11 |** **Supercell and band structure of pure ZnO.**

The structure of pure ZnO is shown in Fig. S11a. In the cell structure, all the Zn and O atoms are identical particles. As shown in Fig. S11b, the band gap of the pure ZnO system is calculated to be 3.40 eV, which is basically consistent with the experimentally obtained value^11^. This result suggests that the calculation parameter setting is reasonable.


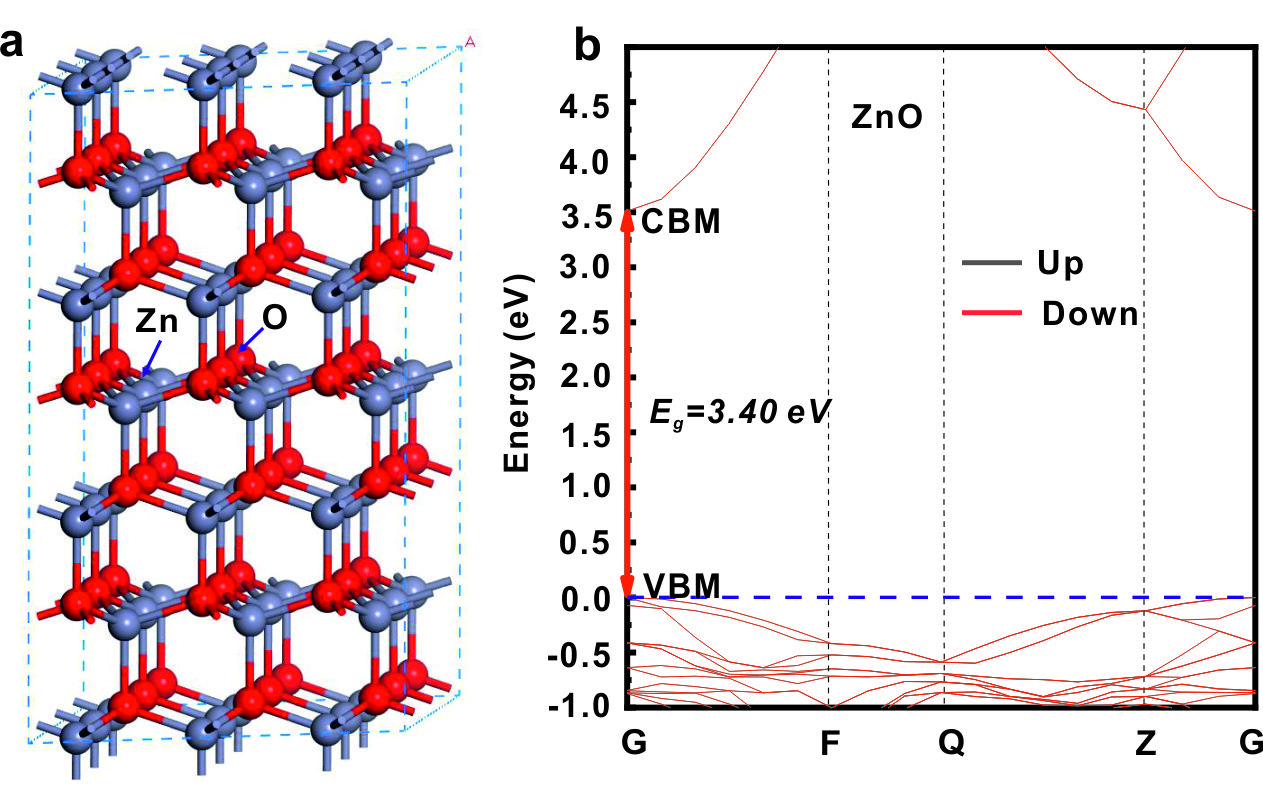


**Fig. S11**  **a** The structure of pure ZnO. **b** Band structure under spin polarization of prue ZnO.

**Table S2** **|** DFT simulation structural parameters^12^

| Max. force (eV·Å^-1^) | 0.05 |
| --- | --- |
| Max stress (Gpa) | 0.1 |
| Max. displacement (Å) | 0.002 |
| Energy (eV·atom^-1^) | 2.0 × 10^–5^ |
| Energy cutoff (eV) | 300 |
| k-point | 2×1×1 |

**References**

1. Jiang, M. M. et al. Wavelength-tunable electroluminescent light sources from individual Ga-doped ZnO microwires. *Small* **13**, 1604034 (2017).
2. Jiang, M. M. et al. An electrically driven whispering gallery polariton microlaser. *Nanoscale* **13**, 5448-5459 (2021).
3. Liu, Y. et al. Electrically excited hot-electron dominated fluorescent emitters using individual Ga-doped ZnO microwires via metal quasiparticle film decoration. *Nanoscale* **10**, 5678-5688 (2018).
4. Chen, A. Q. et al. Electrically driven single microwire-based heterojunction light-emitting devices. *ACS Photonics* **4**, 1286-1291 (2017).
5. Zhu, H. et al. Ultralow-threshold laser realized in zinc oxide. *Advanced Materials* **21**, 1613-1617 (2009).
6. Xu, C. X. et al. Whispering-gallery mode lasing in ZnO microcavities. *Laser & Photonics Reviews* **8**, 469-494 (2014).
7. Zhu, G. Y. et al. Lasing behavior modulation for ZnO whispering-gallery microcavities. *ACS Applied Materials & Interfaces* **4**, 6195-6201 (2012).
8. Qin, F. F. et al. Interfacial control of ZnO microrod for whispering gallery mode lasing. *ACS Photonics* **5**, 2313-2319 (2018).
9. Xu, C. X. et al. Plasmon-enhanced ZnO whispering-gallery mode lasing. *Nano Research* **11**, 3050-3064 (2018).
10. Pei, J. N. et al. Controlled enhancement range of the responsivity in ZnO ultraviolet photodetectors by Pt nanoparticles. *Applied Surface Science* **389**, 1056-1061 (2016).
11. Liu, X. N. et al. Tailoring the third-order nonlinear optical property of a hybrid semiconductor quantum dot-metal nanoparticle: From saturable to fano-enhanced absorption. *Journal of Physical Chemistry Letters* **10**, 7594–7602 (2019).
12. Zheng, H. et al. Monolayer II-VI semiconductors: A first-principles prediction. *Physical Review B* **92**, 115307 (2015).
